# Information borrowing in phase II randomized dose-ranging clinical trials in oncology

**DOI:** 10.1186/s12874-026-02883-4

**Published:** 2026-05-27

**Authors:** Guillaume Mulier, Vincent Lévy, Lucie Biard

**Affiliations:** 1 ECSTRRA Team, UMR 1342 IRSL, Université Paris Cité and INSERM, 1 avenue Claude Vellefaux, Paris, 75010 France; 2 Service de Biostatistique et Informatique Médicale, AP-HP, Hôpital Saint-Louis, 1 avenue Claude Vellefaux, Paris, 75010 France; 3 Clinical research department, Université Sorbonne Paris Nord and AP-HP, Hôpital Avicenne, 125 rue de Stalingrad, Bobigny, 93000 France

**Keywords:** Phase II, Dose-ranging study, Oncology, Borrowing

## Abstract

**Introduction:**

Over the past decades, the advent of new therapeutics such as immunotherapies and targeted therapies has challenged conventional clinical trial designs, such as single-arm studies. Selecting a single dose in phase I trials with short follow-up, typically based solely on toxicity endpoints, has been shown to lead to suboptimal dosing decisions. Consequently, dose optimization is now increasingly encouraged in oncology. This study was motivated by the case of ibrutinib in chronic lymphocytic leukemia, for which the initially approved dose of 420 mg/day, determined using conventional phase I designs based on the maximum tolerated dose, was later shown to achieve comparable response rates at lower doses. This example highlights the potential value of dose-ranging phase II studies in oncology. Assuming that borrowing information across doses can improve statistical power, our objective was to compare several strategies for information borrowing in phase II randomized trials involving multiple doses of the same drug.

**Methods:**

The backbone design considered was the Bayesian Optimal Phase II (BOP2) design, adapted to a multi-arm setting and allowing for co-primary binary endpoints as well as interim analyses. This design relies on a multinomial conjugate model to describe the endpoints within a Bayesian framework, with decision rules for early stopping due to futility and/or toxicity based on posterior probabilities. We adapted and compared several information-borrowing approaches to estimate efficacy and toxicity: (i) power prior, (ii) Bayesian hierarchical modeling, (iii) Bayesian calibrated hierarchical modeling, and (iv) Bayesian logistic regression. These approaches were applied alongside BOP2 decision rules. In addition, a Simon’s two-stage design with added toxicity monitoring was used as a comparator. A simulation study was conducted to evaluate the operating characteristics of the designs, using a hypothetical randomized dose-ranging trial with efficacy and toxicity as co-primary endpoints against reference values.

**Results:**

Our results indicate that the power prior, without dynamic adaptation of the borrowing strength, is unsuitable in this context as it substantially increases the false positive rate. Bayesian hierarchical modeling shrinks estimates toward a common mean, reducing variance but also leading to inflated false positive rates. In contrast, Bayesian logistic regression provides a more balanced trade-off, achieving moderate gains in power while increasing the false positive rates.

**Conclusion:**

In multi-arm trials, the BOP2 design without information borrowing offers stricter control of the false positive rate than borrowing-based approaches when only toxic or futile doses are considered. Nevertheless, Bayesian logistic regression modeling of the dose–toxicity and dose–efficacy relationships, combined with BOP2 decision rules, could be considered as a possible approach for borrowing information in dose-ranging studies with a limited number of doses.

**Supplementary Information:**

The online version contains supplementary material available at 10.1186/s12874-026-02883-4.

## Background

Phase II clinical trials correspond to the second stage of clinical drug development and aim to provide a preliminary assessment of efficacy while ruling out unpromising treatments [1], whereas phase I clinical trials primarily seek to identify an appropriate dose for subsequent phases. In particular, dose selection in phase I trials is often based solely on toxicity under the paradigm that “more is better”. That is, both efficacy and toxicity are assumed to increase monotonically with dose, so that the highest tolerable dose is expected to provide the greatest efficacy [2, 3]. This assumption has historically underpinned the development of cytotoxic chemotherapies in oncology.

Historically, phase II oncology trials have therefore relied on single-dose evaluations, most often using single-arm designs with short-term efficacy endpoints, such as response rate assessed at a fixed time point (*e.g.*, 3 or 6 months) [4].

Since the 2000s, however, this paradigm has been challenged by the emergence of new therapeutic classes, notably targeted therapies [5] and, since the 2010s, immunotherapies including monoclonal antibodies, and cell and gene therapies [6]. These treatments differ substantially from cytotoxic agents in their mechanisms of action and pharmacological properties. In particular, the most tolerable dose is not necessarily the most effective one.

Despite these developments, sequential use of phase I designs followed by single-arm phase II trials remain widely used in oncology [7]. Oncology also exhibits a relatively low success rate of phase III trials compared with other medical fields [8], as well as high rates of dose reductions and treatment discontinuation [9, 10]. Accordingly, several post-approval dose reductions have been reported [11, 12], some following postmarketing requirements or formal post-approval dose optimization [13, 14]. The importance of pre-approval drug dosage optimization has been increasingly emphasized by regulatory agencies [9, 15–17]. In this context, the U.S. Food and Drug Administration launched Project Optimus in 2021 through its Oncology Center of Excellence to promote dose optimization in oncology drug development [17–19], with published guidance in 2024 [16].

As an example, our work was specifically motivated by the case of ibrutinib, a Bruton tyrosine kinase inhibitor approved for mantle cell lymphoma (560 mg/day) and chronic lymphocytic leukemia (420 mg/day) following accelerated approval by the FDA [20] and the European Medicines Agency in 2013 and 2014, respectively. These approvals were based on one phase I trial [21] and two single-arm phase II trials [22, 23] ($n=$ 56, 85 and 111 analyzed patients, respectively). Subsequent randomized trials demonstrated improvements in progression-free survival compared with ofatumumab in chronic lymphocytic leukemia. Further indications were later granted, including Waldenström’s macroglobulinemia [24] and first-line treatment of chronic lymphocytic leukemia following the RESONATE-2 and E1912 trials [25, 26]. Post-marketing experience revealed important toxicity issues, leading to frequent dose reductions due to gastrointestinal, cardiac, or hematological adverse events [27]. Moreover, a pilot study [28] suggested similar pharmacokinetical properties at lower doses. These findings raise the question of whether randomized dose-ranging studies could have identified more appropriate dosing regimens earlier in the drug development process.

Several strategies have been proposed to improve pre-approval dose optimization of anticancer agents. For example, phase I/II designs jointly model toxicity and efficacy in dose-finding studies [29–35]. Another approach consists of selecting a limited number of promising doses in phase I and subsequently conducting randomized dose-ranging phase II trials to identify the optimal dose, rather than focusing solely on the maximum tolerated dose [15, 36]. Such an approach is commonly used in other therapeutic areas [3, 15, 31]. Dose-ranging trials, also referred to as randomized parallel dose–response studies, are recommended by regulatory agencies and may include a control arm, allowing multiple comparisons while controlling the overall type I error rate [37]. Alternatively, they may rely on decision rules comparing each dose to predefined reference values [31, 38], without including a control arm [16].

In basket oncology phase II trials, where multiple tumor types are treated with the same treatment based on a shared biological mechanism or molecular target, information-borrowing approaches have been proposed to improve operating characteristics under limited sample sizes [39, 40]. These approaches rely on the assumption of partial similarity in efficacy and toxicity across indications, typically justified by the shared treatment. The Bayesian hierarchical model (BHM) was among the first approaches proposed for this purpose, treating treatment effects as exchangeable draws from a common distribution [41]. Extensions of this framework include variance calibration [42] and models allowing for partial exchangeability, such as EXNEX model [39, 40]. In dose-ranging studies, however, information borrowing across doses has been less extensively studied and has mainly relied on dose–response modeling approaches mainly for efficacy, such as MCP-Mod [37] or Bayesian dynamic linear models [43]. To our knowledge, borrowing strategies inspired by basket trial methodology have rarely been applied to randomized dose-ranging studies [44–48].

Motivated by the Ibrutinib case study, we hypothesized that borrowing information across randomized dose levels could improve the operating characteristics of phase II dose-ranging designs. The objective of this work was therefore to develop and compare several dose-ranging designs incorporating information borrowing in the context of pre-approval oncology trials. We considered phase II randomized designs including multiple dose levels without a control arm, where decisions are based on comparisons with prespecified efficacy and toxicity thresholds rather than direct between-arm comparisons. Efficacy and toxicity were modeled as co-primary endpoints, and interim analyses with early stopping rules for futility and toxicity were incorporated.

The remainder of the article is organized as follows. Methods section describes the considered designs and statistical models and presents the simulation study. Results section illustrates the methods using the ibrutinib case study simulations, and Discussion and Conclusion sections provides a discussion and concluding remarks.

## Methods

We considered the design of a trial in which patients were equally randomized across *K* experimental arms comparing *K* dose levels. Efficacy and toxicity were defined as co-primary binary endpoints, and *J* analyses were planned. Reference values for efficacy and toxicity rates, denoted $\phi _{\text {eff}}$ and $\phi _{\text {tox}}$, respectively, were specified. These values represent thresholds below or above which a treatment is considered futile or excessively toxic, respectively, similarly to null hypotheses for inefficacy and toxicity, and could be elicited from clinical expertise.

Let $\textbf{Y}_k = (Y_{k,1}, Y_{k,2}, Y_{k,3}, Y_{k,4})$ denote the vector of random variables representing the counts of the four possible individual outcomes in arm *k*, with $k \in \{1, \ldots , K\}$. Specifically, $Y_{k,1}$ denotes the number of patients experiencing both response and toxicity, $Y_{k,2}$ the number of patients with response but no toxicity, $Y_{k,3}$ those with toxicity but no response, and $Y_{k,4}$ those with neither response nor toxicity. Let $D_{n_j,k} = \{n_{j,k}, x_{j,k,\text {eff}}, x_{j,k,\text {tox}}\}$ denote the observed data in arm *k* at analysis *j*, where $n_{j,k}$ is the number of patients enrolled in arm *k* at analysis *j*, $x_{j,k,\text {eff}}$ the number of observed responses, and $x_{j,k,\text {tox}}$ the number of observed toxicities.

We therefore considered several trial designs allowing for co-primary endpoints, interim analyses, and randomization without a control arm. Reference designs without information borrowing (BOP2 design and Simon’s two-stage design with toxicity monitoring sections) were compared with approaches incorporating information sharing across treatment arms (Information borrowing across arms section).

### BOP2 design

The Bayesian Optimal Phase II design (BOP2) was initially proposed as a single-arm group-sequential design for phase II clinical trials allowing for multiple endpoints. It has since been extended to randomized settings [49, 50].

In a randomized trial with multiple experimental arms analyzed on an intent-to-treat basis, the extended BOP2 design models the outcome counts $\textbf{Y}_k$ in each arm separately using a conjugate multinomial distribution with probability vector $\boldsymbol{\theta }_k = (\theta _{k,1}, \theta _{k,2}, \theta _{k,3}, \theta _{k,4})$. A Dirichlet prior with parameters $(\pi _{0,1}, \pi _{0,2}, \pi _{0,3}, \pi _{0,4})$ is assumed for $\boldsymbol{\theta }_k$, representing outcome probabilities under the inefficacy and toxicity hypotheses as elicited from clinical expertise. The corresponding conjugate posterior distribution of $\boldsymbol{\theta }_k$ is also Dirichlet.

At a given interim analysis *j*, with $n_{j,k}$ patients accrued in each arm *k*, the marginal posterior distributions of the efficacy and toxicity probabilities are given by $p_{j,k,\text {eff}} \mid D_{n_j,k} \sim \textrm{Beta}\left( \pi _{0,1} + \pi _{0,2} + x_{j,k,\text {eff}},\pi _{0,3} + \pi _{0,4} + n_{j,k} - x_{j,k,\text {eff}}\right)$ and $p_{j,k,\text {tox}} \mid D_{n_j,k} \sim \textrm{Beta}\left( \pi _{0,1} + \pi _{0,3} + x_{j,k,\text {tox}},\pi _{0,2} + \pi _{0,4} + n_{j,k} - x_{j,k,\text {tox}}\right)$.

At each analysis *j* (interim and final), arm *k* with $n_{j,k}$ recruited patients can be stopped early for futility and/or toxicity if:

$$\begin{aligned} \left\{ \begin{array}{l} Pr(p_{j,k,\text {eff}}\le \phi _\text {eff}|D_{n_j,k)}>C_{n_j}\\ Pr(p_{j,k,\text {tox}}>\phi _\text {tox}|D_{n_j,k})>C_{n_j} \end{array}\right. \end{aligned}$$

Here, $\Pr (p_{k,\dots } \mid D_{n_j,k})$ denotes posterior probabilities derived from the marginal distributions described above, and $C_{n_j}$ is a decision threshold that varies over the course of the trial. This threshold is defined as $C_{n_j} = 1 - \lambda \left( \frac{n_{j,k}}{N}\right) ^{\gamma }$, where $n_{j,k}$ is the number of patients per arm at interim analysis *j* in arm *k*, *N* is the maximum planned sample size per arm, and $\lambda$ and $\gamma$ are tuning parameters selected via grid search. These parameters are chosen to control a prespecified family-wise error rate (FWER) across the *K* arms (corresponding to incorrectly declaring at least one arm promising when all arms are futile or overly toxic) while maximizing power under the least favourable configuration (defined as the scenario in which exactly one arm is efficacious and safe while all others are futile or toxic [51]). In the remainder of this article, this design is referred to as the mBOP design (“multi-arm BOP”).

### Information borrowing across arms

To enable information borrowing across treatment arms, we considered several Bayesian approaches to adapt the mBOP design described above (BOP2 design section). These approaches were used to compute the posterior distributions of efficacy and toxicity in each arm while incorporating information from the other arms. All proposed adaptations were implemented in conjunction with the mBOP decision rules described previously.

#### Power prior approach

The power prior approach was originally proposed by Ibrahim and Chen to incorporate information from historical data when analyzing a current dataset [52].

Briefly, a power prior is defined as the product of a chosen prior distribution and the likelihood of historical data raised to a power parameter $\alpha _0$. This power prior is then combined with the likelihood of the current data to obtain the posterior distribution. Multiple historical data sources can be incorporated by taking the product of their likelihoods, each raised to the power $\alpha _0$. The parameter $\alpha _0$, with $0 \le \alpha _0 \le 1$, controls the amount of borrowing: $\alpha _0 = 0$ corresponds to ignoring historical information entirely, whereas $\alpha _0 = 1$ corresponds to a pooled analysis of historical and current data.

We applied the power prior approach to estimate efficacy and toxicity rates in each arm at a given interim analysis *j*, allowing information borrowing across arms, as multiple doses of the same drug were randomized in the trial. Under the Dirichlet-multinomial conjugate framework of the mBOP design, the posterior distributions of the marginal efficacy and toxicity probabilities in arm *k* can be written as:

$$\begin{aligned}&p_{j,k,\text {eff}}|D_{n_j,k}\sim \text {Beta}(\pi _{0,1}+\pi _{0,2}+\alpha _0\sum \nolimits _{l=1;l \ne k}^{K} x_{j,l,\text {eff}}+x_{j,k,\text {eff}},\\&\pi _{0,3}+\pi _{0,4}+\alpha _0\sum \nolimits _{l=1;l \ne k}^{K} (n_{j,l}-x_{j,l,\text {eff}})+n_{j,k}-x_{j,k,\text {eff}}) \end{aligned}$$

and

$$\begin{aligned}&p_{j,k,\text {tox}}|D_{n_j,k}\sim \text {Beta}(\pi _{0,1}+\pi _{0,3}+\alpha _0\sum \nolimits _{l=1;l \ne k}^{K} x_{j,l,\text {tox}}+x_{j,k,\text {tox}},\\&\pi _{0,2}+\pi _{0,4}+\alpha _0\sum \nolimits _{l=1;l \ne k}^{K} (n_{j,l}-x_{j,l,\text {tox}})+n_{j,k}-x_{j,k,\text {tox}}). \end{aligned}$$

The power parameter $\alpha _0$ was treated as fixed and specified beforehand the trial. In our analyses, it was set to $\alpha _0 = 0.5$, representing a compromise between increased power and control of the FWER.

This design is referred to as powBOP hereafter.

#### Bayesian hierarchical model

Bayesian hierarchical modeling (BHM) has been proposed to enable information borrowing across subpopulations in phase II oncology trials [41]. We applied BHM separately to the efficacy and toxicity endpoints. Under the BHM framework, the true efficacy and toxicity probabilities in each of the *K* arms are assumed to arise from a common distribution, respectively, such that:

$$\begin{aligned} (x_{1,\cdot },\dots ,x_{K,\cdot })&\sim \text {Binomial}((n_1,\dots ,n_K),(p_{1,\cdot },\dots ,p_{K,\cdot })) \\ \text {logit}((p_{1,\cdot },\dots ,p_{K,\cdot }))&\sim \mathcal {N}(\mu _\cdot ,\sigma _\cdot ^2) \end{aligned}$$

The Gaussian distribution $\mathcal {N}(\mu _\cdot ,\sigma _\cdot ^2)$ was used to model the heterogeneity across arms, where $\mu _\cdot$ represents the overall mean logit response and $\sigma _\cdot ^2$ controls the degree of information borrowing between arms for the logit of efficacy and toxicity probabilities, $p_{\text {eff}}$ and $p_{\text {tox}}$, respectively. When $\sigma _\cdot ^2 = 0$, all arms share the same mean, resulting in complete pooling; conversely, as $\sigma _\cdot ^2 \rightarrow \infty$, no borrowing occurs and the analysis approaches that of independent arms. This design is referred to as hBOP hereafter.

A known limitation of Bayesian hierarchical models is that the heterogeneity hyperparameter $\sigma _\cdot ^2$ cannot be reliably estimated when the number of groups (here, treatment arms) is small [53]. Because dose-ranging phase II trials typically involve a limited number of arms, we also considered the calibrated BHM approach proposed by Chu and Yuan [42]. In this framework, the hyperparameter $\sigma _\cdot ^2$ is fixed prior to model fitting and computed as a function of a measure of between-arm homogeneity.

Specifically, $\sigma _\cdot ^2$ is defined as

$$\begin{aligned} \sigma _\cdot ^2 = e^{a + b \times \log (T_\cdot )}, \end{aligned}$$

where $T_\cdot$ is a heterogeneity measure defined using a $\chi ^2$ statistic:

$$\begin{aligned} T_\cdot = \sum \limits _{k=1}^K \frac{(O_{0k_\cdot } - E_{0k_\cdot })^2}{E_{0k_\cdot }} + \sum \limits _{k=1}^K \frac{(O_{1k_\cdot } - E_{1k_\cdot })^2}{E_{1k_\cdot }}. \end{aligned}$$

Here, $O_{0k_\cdot }$ and $E_{0k_\cdot }$ denote the observed and expected numbers of non-responses or non-toxicities in arm *k*, respectively, while $O_{1k_\cdot }$ and $E_{1k_\cdot }$ denote the observed and expected numbers of responses or toxicities. Chu and Yuan [42] propose a three-step simulation-based procedure to calibrate the parameters *a* and *b* under scenarios of efficacy/non-toxicity and inefficacy/toxicity. This design is referred to as cbhmBOP hereafter. These 2 designs were estimated via Markov Chain Monte Carlo (MCMC) with STAN.

#### Bayesian logistic regression

As a form of information borrowing across arms, we considered a two-parameter Bayesian logistic regression model, originally proposed by Neuenschwander *et al.*, to model the dose–toxicity relationship across dose levels in phase I dose-escalation trials [54]. Let $d_k$ denote the dose administered in arm *k*, and $d^*$ a reference dose. We extended this approach to model both dose–efficacy and dose–toxicity relationships across arms. The model can be written as:

$$\begin{aligned} \text {logit}(p_{k,\cdot }) = \alpha _\cdot + \beta _\cdot \frac{d_k}{d^*}, \end{aligned}$$

where $(\alpha _\text {eff}, \beta _\text {eff})$ and $(\alpha _\text {tox}, \beta _\text {tox})$ are the parameters governing the efficacy and toxicity models, respectively. This design is hereafter referred to as log1BOP.

To enforce a monotonic increase of efficacy and toxicity with dose, we further considered a constrained version of the model by imposing $\beta _\cdot> 0$ for both endpoints. This design is referred to as log2BOP in the following. The Bayesian logistic regressions were estimated via MCMC with STAN.

### Simon’s two-stage design with toxicity monitoring

Simon’s two-stage design is widely used in early phase II oncology trials, as it allows a limited sample size with one interim analysis ($J=2$) and a futility stopping rule, which is consistent with the objective of drug selection [55]. The design is based on the binomial enumeration of the number of responses under the null and alternative hypotheses in order to optimize stopping boundaries while controlling type I and II error rates. The arm is stopped at the interim analysis for futility if there are $r_1$ or fewer responses observed in arm *k*, and at the final analysis, the arm is deemed efficacious if there are more than *r* responses. The design is said to be optimal when the decision thresholds and sample sizes at each analysis are optimized to minimize the expected number of patients under the null hypothesis [55]. In a multi-arm phase II design, a multiplicity correction may be applied (*e.g.*, Bonferroni correction), and the design may be applied independently in each arm.

For comparative purposes with the other approaches, we added a Bayesian toxicity monitoring component, in which toxicity was modeled using a Bayesian beta-binomial model [56, 57]. Under this framework, the arm is stopped for toxicity at analysis *j* if $Pr(p_{j,k,\text {tox}}>\eta \mid D_{n_j,k})>\tau$, where $\eta$ and $\tau$ are optimized through simulations (see Appendix file 1, Section 1). This design is denoted Simon+TM hereafter.

### Simulation settings

**Fig. 1:**
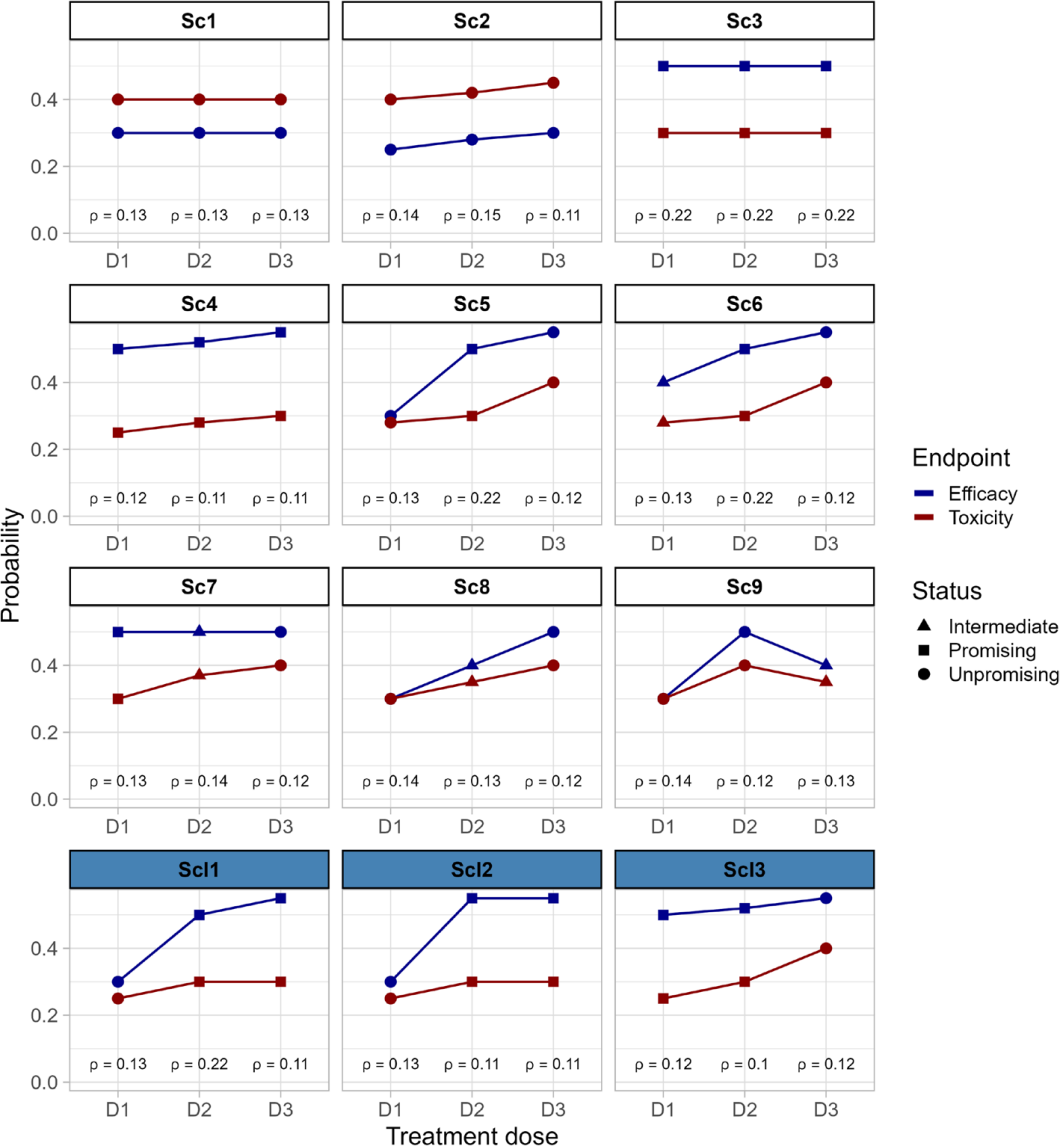
Simulation scenarios: probability of efficacy and toxicity for each dose (highlighted: scenarios ScI1, ScI2 and ScI3 representative of plausible situation for the ibrutinib use case). The correlation presented are the correlation coefficients between efficacy and toxicity

Operating characteristics of the studied designs were evaluated through Monte Carlo simulations mimicking plausible scenarios for the evaluation of ibrutinib (Fig. 1). Specifically, the reference values corresponding to inefficacy and toxicity were $\{\pi _\text {eff}=0.30;\pi _\text {tox}=0.40\}$ and those corresponding to desirable efficacy and acceptable toxicity were $\{\pi _\text {eff}=0.50;\pi _\text {tox}=0.30\}$. For each scenario, a slightly positive correlation between efficacy and toxicity across dose levels was assumed.

Specifically, trials with $K=3$ arms corresponding to three hypothetical doses of ibrutinib (140, 280 and 420 mg per day) were simulated under various scenarios. In scenarios 1 and 2, all three doses had efficacy and toxicity rates consistent with the null hypothesis (i.e., futile and toxic), with dose–response relationships that were either strictly flat or slightly increasing, respectively (Fig. 1). Similarly, in scenarios 3 and 4, all three doses were efficacious and safe (alternative hypothesis), with flat or slightly increasing dose–response relationships. In scenario 5, dose–response curves were more pronounced, with only the second dose being promising, while the lowest dose was inefficacious and the highest dose was toxic. Scenarios 6 and 7 both included a single promising dose and one dose with intermediate efficacy or toxicity, that is, slightly below the desirable efficacy level or above the acceptable toxicity level, respectively. Scenario 8 featured increasing dose–response relationships from an inefficacious lowest dose to a toxic highest dose, with a middle dose showing moderate efficacy and toxicity. Scenario 9 was a non monotonic scenario with inverted U-shaped dose-efficacy and dose-toxicity relationship with no promising dose. In addition, three clinically plausible scenarios for phase II dose-ranging trials of ibrutinib were considered: scenario I1, in which the lowest dose was inefficacious and the two higher doses were promising; scenario I2 which is similar to scenario I1 except that the dose-efficacy relationship is not monotonic with efficacy and toxicity plateauing after dose 2; and scenario I3, in which the two lowest doses were promising while the highest dose was too toxic. The detail of the probabilities used to simulated the scenarios are presented in Additional file 1.

Hyperparameters of the mBOP design and sample sizes were calibrated to ensure a maximum FWER of 10% and a minimum power of 80% in each arm. The resulting hyperparameters, calibrated by grid search under an inefficacy/toxicity hypothesis of $\{\pi _\text {eff}=0.30;\pi _\text {tox}=0.40\}$ and an efficacy/acceptable toxicity hypothesis of $\{\pi _\text {eff}=0.50;\pi _\text {tox}=0.30\}$, were $\lambda = 0.76$ and $\gamma = 1.31$, corresponding to an estimated FWER of 9.13% and an arm-power of 80.64%, over 10,000 trial simulations. A maximum sample size of 58 patients per arm, with one interim analysis in each arm, at 29 patients, was defined, in between the sample sizes of the inital Ibrutinib trials, phase I and single-arm phase II studies [21–23]. This threshold was also applied to hBOP, cbhmBOP, log1BOP and log2BOP because of the computational burden of optimizing such threshold with models estimated by MCMC.

For the BHM and CBHM approaches, weakly informative skeptical priors centered on the null hypotheses were used for the mean efficacy and toxicity rates: $\mu _\text {eff} \sim N(-0.85,2.5^2)$, corresponding to a prior mean efficacy rate of 0.30 with a 95% credible interval of [0.00–0.98], and $\mu _\text {tox} \sim N(-0.41,2.5^2)$, corresponding to a prior mean toxicity rate of 0.40 with a 95% credible interval of [0.00–0.99]. For the BHM approach, half-normal priors were used for $\sigma _\text {eff}$ and $\sigma _\text {tox}$, i.e., *N*(0, 1) with support restricted to positive values. For the CBHM approach, $\sigma _\text {eff}$ and $\sigma _\text {tox}$ were separately defined according to the procedure of Chu and Yuan [42], with calibrated parameters $a=-0.94$ and $b=2.65$ for efficacy, and $a=-2.43$ and $b=6.70$ for toxicity.

For Bayesian logistic regression, similar weakly informative priors were used for the intercept parameters (mean efficacy and toxicity rates for hypothetical dose 0): $\alpha _\text {eff} \sim N(-0.85,2.5^2)$ and $\alpha _\text {tox} \sim N(-0.41,2.5^2)$. Priors for the slope parameters were chosen to reflect monotonic dose–response and dose-toxicity relationships: $\beta _\text {eff} \sim N(0.42,2.5^2)$, corresponding to an odds ratio (OR) of 1.52 per dose unit increase (95% credible interval: 0.01-204.37), and $\beta _\text {tox} \sim N(0.22,2.5^2)$, corresponding to an OR of 1.25 (95% credible interval: 0.01-167.32).

For the Simon+TM design, futility boundaries were set to $r_1=11$ and $r=20$, and the toxicity monitoring rule stopped arm *k* if $Pr(p_{k,\text {tox}}>0.4 \mid D_{n,k})>0.5$, assuming a uniform prior $\text {Beta}(1,1)$ (see Additional file 1 for details on design parameterization).

For each scenario, 5,000 simulated trials were generated to evaluate the probability of concluding to efficacy with acceptable toxicity (promising treatment), the average sample size, and the probability of early stopping. All analyses were conducted using R version 4.3.0.

## Results

In the following, doses will be refered to as arms in the text. Arm 1 is the lowest dose, arm 2 the middle dose and arm 3 the highest dose.

### Scenarios 1 to 4: FWER and power

**Fig. 2:**
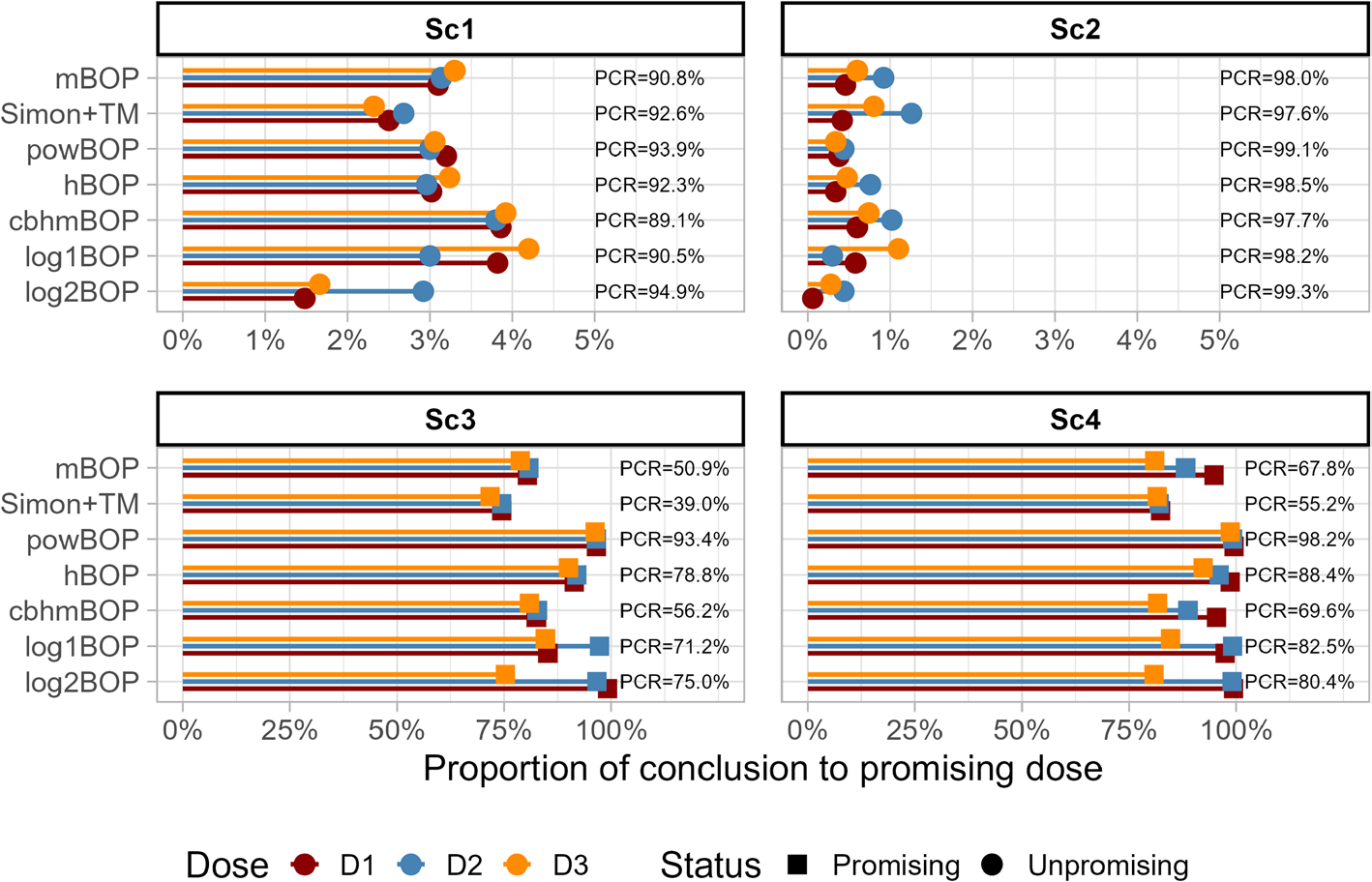
Proportion of trials concluding in favor of a promising treatment within each arm for scenarios 1 to 4, over 5,000 simulations. The symbol at the end of the bar represents the true status of the dose (promising, intermediate, unpromising). Doses are in increasing order from bottom to top for each design. PCR stands for probability of correct recommendation, that is recommending exactly the promising doses in a given scenario

Table 1 reports the estimated probability of concluding that at least one arm is promising at the end of the trial across scenarios 1 to 4 and Fig. 2 shows the results for each arm separately for scenarios 1 to 4.

**Table 1:** Proportion of trials with at least one dose deemed promising for each studied design in scenarios 1 to 4

Scenario	mBOP	Simon+TM	powBOP	hBOP	cbhmBOP	log1BOP	log2BOP
1	9.2	7.4	6.1	7.7	10.9	9.5	5.1
2	2.0	2.4	0.9	1.5	2.3	1.8	0.7
3	99.3	98.2	98.7	98.9	99.0	99.2	99.6
4	99.9	99.4	99.8	99.8	99.8	99.8	99.9

The FWER at the trial level was controlled at the prespecified 10% level, except for cbhmBOP, which exhibited a slightly higher rate (10.9%) of concluding to a promising treatment in scenario 1. At the arm level, the proportions of conclusions in favor of a promising treatment were similar across methods and remained below 5% in all three arms in scenario 1 and below 2% in scenario 2 (Fig. 2).

In scenarios 3 and 4, corresponding to desirable settings for all three arms (alternative hypothesis), the estimated probability of concluding to at least one promising arm was close to 100% for all methods. At the arm level, in scenario 3, power ranged from 71.7% with Simon+TM to 99.2% with log2BOP for arm 1, and, in scenario 4, from 80.8% with log2BOP for arm 3 to 99.5% with powBOP. Among methods incorporating information borrowing, only powBOP and hBOP consistently exhibited higher power than mBOP across arms. Power was similar across arms in scenarios 3 and 4 for most methods, except for log1BOP and log2BOP, which showed higher power in arms 1 and 2 than in arm 3, with differences ranging from 12% to 24%.

### Scenarios 5 to 9

**Fig. 3:**
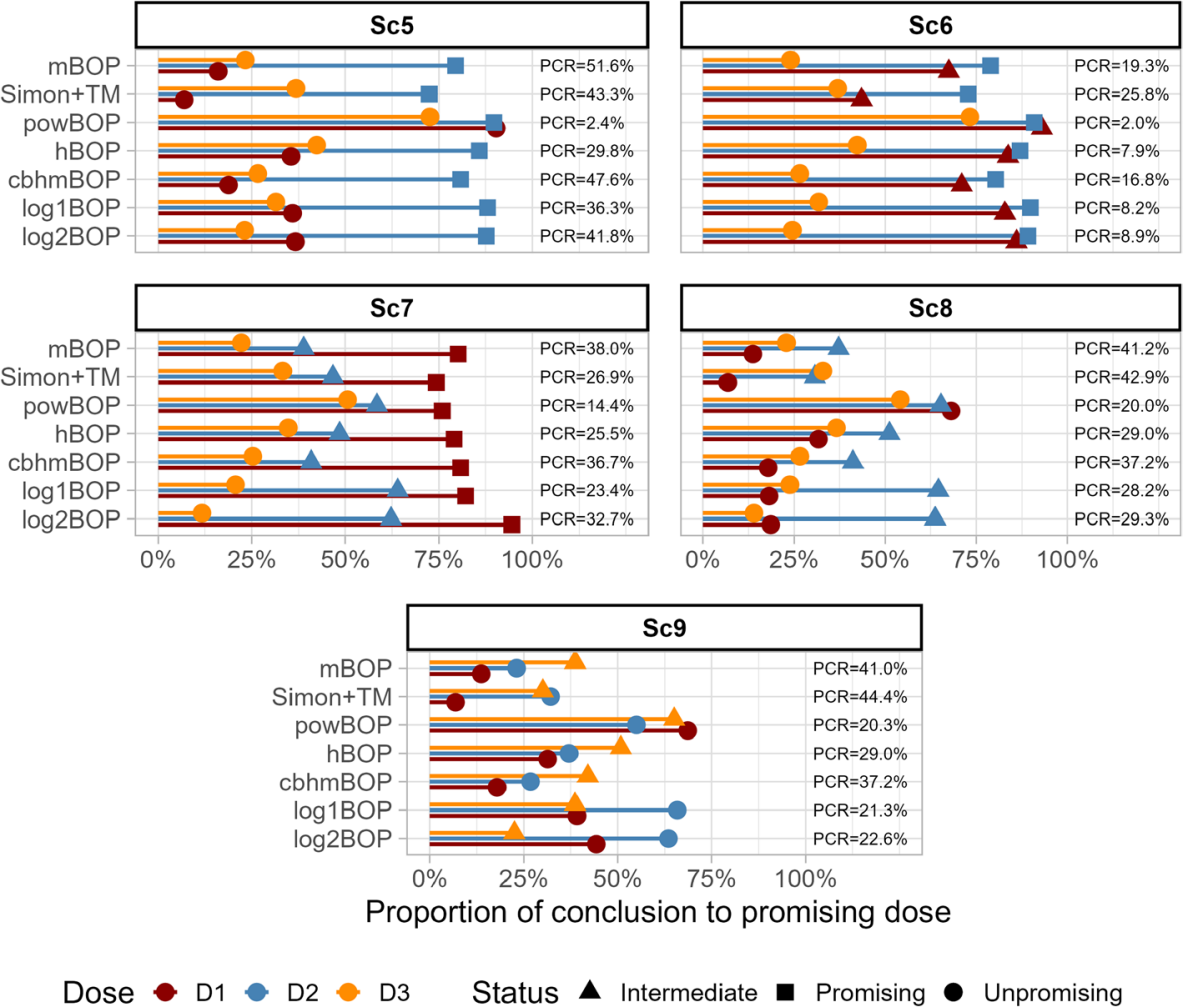
Proportion of trials concluding in favor of a promising treatment within each arm for scenarios 5 to 9, over 5,000 simulations. The symbol at the end of the bar represents the true status of the dose (promising, intermediate, unpromising). Doses are in increasing order from bottom to top for each design. PCR stands for probability of correct recommendation, that is recommending exactly the promising doses in a given scenario

Results in scenarios 5 to 9, representing situations departing from the planning hypotheses on efficacy and/or toxicity, are presented in Fig. 3 and in Table S2 in the Additional file 1.

In these scenarios, borrowing methods were generally outperformed by mBOP and Simon+TM in correctly recommending exactly the true promising dose(s) at the end of the trial (probability of correct recommendation (PCR)). The best PCR rose at most at 51.6% (mBOP) in scenario 5, where only arm 2 was promising whereas arm 1 was clearly inefficacious and arm 3 toxic, while scenario 6, which was similar to scenario 5 but with intermediate efficacy in arm 1, appeared the most challenging with at most 25.8% PCR (Simon+TM).

In terms of false positive conclusions, all methods had rates rising above the desired threshold for FWER across arms (10%), and also in each arm individually, except for Simon+TM which controlled the false positive rate at 7.0% in truly inefficacious arms (arm 1 in scenarios 5, 8 and 9). In truly toxic arms in monotone relationships across arms (*e.g.*, arm 3 in scenarios 5 to 8), logistic regression approaches and cbhmBOP had similar false positive rates as non-borrowing mBOP (ranging from around 13% in arm 3 of scenarios 7 and 8 with log2BOP to about 33%), while those of Simon+TM, hBOP were slightly higher, from 32.2% to 42.4%. These results were observed in a similar manner when considering early stopping decisions in truly inefficacious arms: Simon+TM then cbhmBOP and mBOP had greater early stopping rates than the other borrowing methods. In truly toxic arms within a monotone scenario, Simon+TM outperformed all other designs in early stopping, while the logistic regression methods were the best performing of borrowing approaches. Borrowing with the power prior approach (powBOP) exhibited the highest false positive rates, overall across scenarios; it rose up to 73.2% for a toxic arm in scenario 6 and 90.4% for an inefficacious arm in scenario 5. For all methods, the false positive rates were greater in the case of intermediate arms than in clearly toxic or inefficacious arms. For instance, in scenario 6, false positive rate in arm 1 (safe but with intermediate efficacy), ranged from 43.5% (method Simon+TM) to 93.0% (method powBOP), while it ranged from 23.5% (method mBOP) to 73.2% (method powBOP) in arm 3 with clearcut toxicity.

Consistently, in truly promising arms, borrowing approaches showed slightly greater power than mBOP, cbhmBOP and Simon+TM. For instance in scenario 5 arm 2 (72.4% to 80.8% vs 85.8% to 89.8% for the other methods), and in scenario 6 arm 2 (72.8% to 80.3% vs 87.0% to 90.9% for the other methods). Of note, in scenario 7 arm 1, only logistic regression approaches gained power compared to mBOP and cbhmBOP. Overall, not considering the power prior approach which had the greatest false positive rates, borrowing with logistic regression approaches showed the greatest power (*e.g.*, scenario 7). Mean number of patients followed early stopping rates.

In the specific case of scenario 9 with inverted U-shaped dose-response relationships for efficacy and toxicity, beside powBOP, the logistic regression methods had the worst results in terms of false positive rates, with estimates led by the models’ assumptions, notably in arm 3 (Additional file 1, Figures S4 and S5).

### Scenarios I1, I2 and I3: Ibrutinib use case

**Fig. 4:**
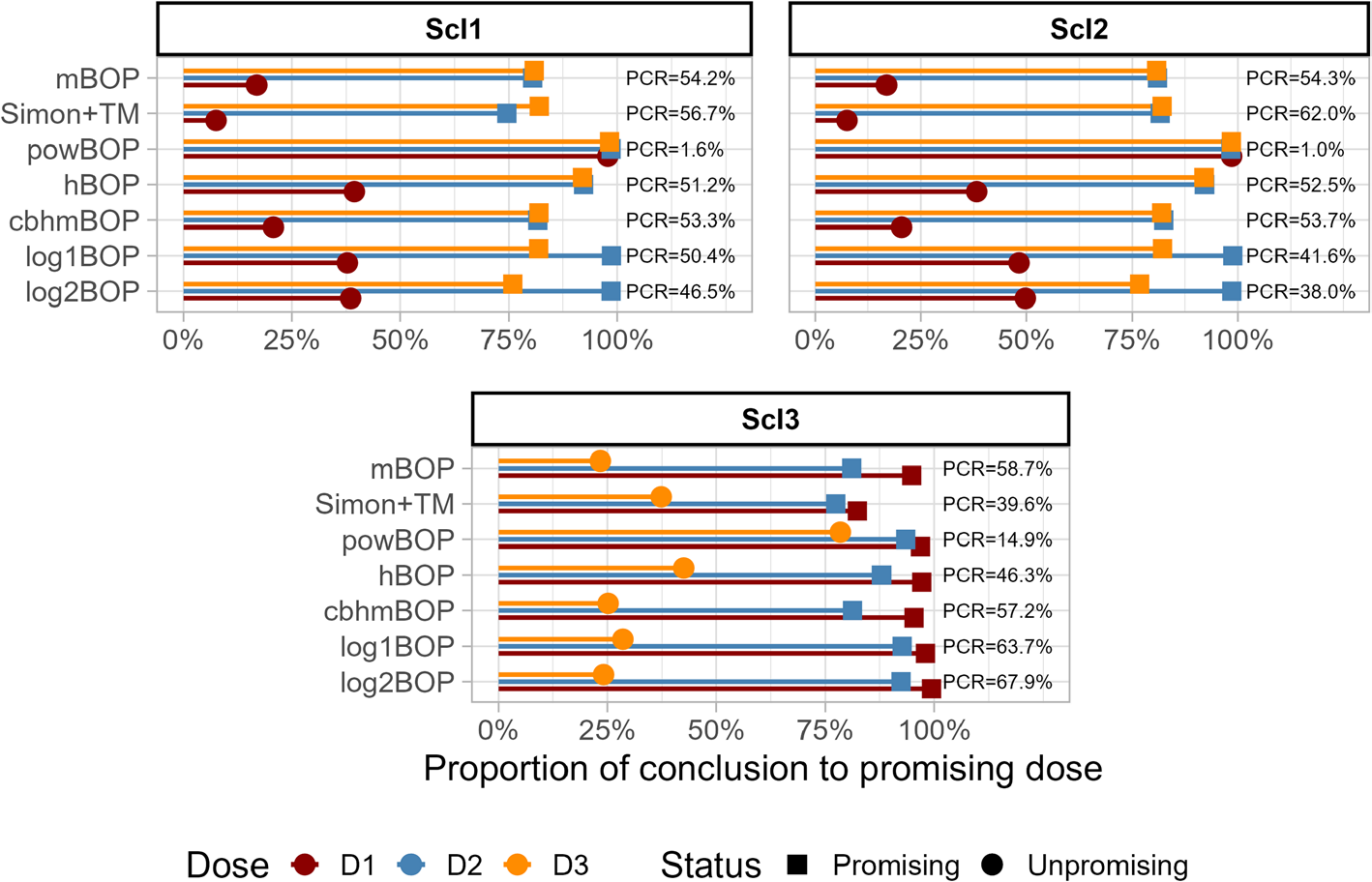
Proportion of trials concluding in favor of a promising treatment within each arm for scenarios I1, I2 and I3, over 5,000 simulations. The symbol at the end of the bar represents the true status of the dose (promising, intermediate, unpromising). Doses are in increasing order from bottom to top for each design. PCR stands for probability of correct recommendation, that is recommending exactly the promising doses in a given scenario

Results on the proportion of conclusions in favor of a promising treatment in each arm are presented in Fig. 4.

In the scenario where the lowest arm was inefficacious (Sc I1), all methods exhibited increased false positive rates in arm 1, ranging from 16.9% for mBOP to 97.8% for powBOP, except for Simon+TM (7.5%). mBOP provided correct conclusions in arms 2 and 3 in 80.5% and 80.9% of cases, respectively, whereas information borrowing methods ranged from 82% to 99%, with higher rates in arm 2 than in arm 3 for Bayesian logistic regression approaches. Simon+TM showed performance comparable to mBOP for selecting arm 3 as promising but had a lower selection rate for arm 2. Regarding early stopping, 33.2% of trials correctly had arm 1 stopped early with mBOP. The proportion of correct early stopping in arm 1 was lower for powBOP (0.5%), hBOP (14.2%), log1BOP (20.0%), and log2BOP (20.4%), comparable for cbhmBOP (33.5%), and higher for Simon+TM (88.1%). For arms 2 and 3, mBOP showed erroneous early stopping rates of 3.1% and 2.9%, respectively. Simon+TM and cbhmBOP exhibited higher erroneous early stopping rates, whereas powBOP and hBOP showed lower rates in these arms. Bayesian logistic regression methods showed lower erroneous stopping in arm 2 but higher in arm 3. Consistently, the mean number of patients in arm 1 was 48.4 for mBOP. This number was higher for powBOP (57.8), hBOP (53.9), log1BOP (52.2), and log2BOP (52.1); similar for cbhmBOP (48.3); and lower for Simon+TM (32.5).

In scenario I2, which is similar to scenario I1 but with plateau for efficacy and toxicity, all methods exhibited increased false positive rates in arm 1, ranging from 16.9% for mBOP to 98.5% for powBOP, except for Simon+TM (7.5%). mBOP provided correct conclusions in arms 2 and 3 in 81.0% and 80.8% of cases, respectively, whereas information borrowing methods ranged from 76% to 99%, with higher rates in arm 2 than in arm 3 for Bayesian logistic regression approaches. Simon+TM performed comparably to mBOP for selecting arms 2 and 3 as promising. Regarding early stopping, mBOP had arm 1 correctly stopped early 33.2% of trials. The proportion of correct early stopping in arm 1 was lower for powBOP (0.3%), hBOP (14.8%), log2BOP (15.3%), and log1BOP (15.5%), comparable for cbhmBOP (33.6%), and higher for Simon+TM (88.1%). For arms 2 and 3, mBOP showed erroneous early stopping rates of 2.9% and 3.0%, respectively. Simon+TM and cbhmBOP exhibited higher erroneous early stopping rates, whereas powBOP and hBOP showed lower rates in these arms. Bayesian logistic regression methods showed lower erroneous stopping rates in arm 2 but higher in arm 3.

In scenario I3, corresponding to a dose-optimization setting where the highest arm is toxic (a situation that may occur pre- or post-approval), no method controlled the false positive rate in arm 3 below the prespecified 10% level, with values ranging from 23.4% for mBOP to 78.5% for powBOP (Fig. 4). mBOP yielded correct conclusions in arms 1 and 2 in 94.8% and 81.1% of cases, respectively, whereas information borrowing methods ranged from 95.4% to 99.4% in arm 1 and from 81.3% to 93.4% in arm 2. Simon+TM showed lower power and a higher false positive rate than mBOP in this scenario. Regarding early stopping, 23.3% of trials had arm 3 correctly stopped early with mBOP. This proportion was lower for powBOP (3.8%) and hBOP (14.5%), comparable for log1BOP (25.2%), and higher for cbhmBOP (29.4%), log2BOP (30.6%), and Simon+TM (52.9%). For arms 1 and 2, mBOP exhibited erroneous early stopping rates of 0.7% and 3.2%, respectively. Simon+TM and cbhmBOP had higher erroneous stopping rates, whereas the other methods showed similar or lower values. The mean number of patients in arm 3 was 51.2 for mBOP. This number was higher for powBOP (56.9) and hBOP (53.8), comparable for log1BOP (50.7), and lower for cbhmBOP (49.5), log2BOP (49.1), and Simon+TM (42.7).

The percentages of correctly recommending the promising arms in both scenarios, that is, concluding in favor of the truly promising arms and against futile or toxic arms, are presented in Fig. 4.

Of note, the Bayesian logistic model can also be specified using $\log \left( \frac{d_k}{d^*}\right)$ as the dose covariate. This parameterization led to improved performance in scenario I1 but worse performance in scenario I3 (results not shown).

## Discussion

We compared several Bayesian models to compute posterior probabilities of efficacy and toxicity within the stopping rules of the BOP2 design [50] incorporating information sharing across arms in the setting of dose-ranging randomized trials in oncology. A simulation study was conducted to evaluate these approaches in plausible scenarios inspired by the use case of ibrutinib in chronic lymphocytic leukemia.

We selected the BOP2 design because it allows comparison of multiple arms against a reference value [49] and incorporates early stopping rules for futility and/or toxicity. This feature is particularly relevant, as when a treatment is truly effective and not overly toxic, recruitment continues until the maximum planned sample size is reached, allowing more data to be collected on promising doses before phase III evaluation. The choice of a fixed reference for comparison, based on historical data which may not be fully comparable to the targeted trial population, may however be questioned. Alternatively, a beta distribution could be specified for the historical reference, instead of a fixed value, and posterior distributions could be approximated [58] for comparisons with the experimental arms. Controlled designs, using the standard of care for instance in a concurrent control arm, can also be implemented within the BOP2 framework [59]. Another limitation lies in the fact that the BOP2 design relies on binary endpoints, whereas, in oncology settings, severe toxicity or disease progression may enventually preclude the complete observations of such endpoints, due to treatment or trial discontinuation and patients being considered unevaluable. Nevertheless, in randomized settings, it is usually recommended that patients be followed-up despite intercurrent events, to allow endpoints evaluation on the randomized sample, following a intent-to-treat approach, with missing data possibly handled by imputation (under specific assumptions).

Overall, our results illustrate that static borrowing using a standard power prior method is not well suited for information sharing across concurrent randomization arms [60], resulting in increased false positive rates. Borrowing information across all arms tended to smooth estimates and resulted in behavior close to that of a pooled analysis. Simon’s design combined with Bayesian toxicity monitoring (Simon+TM), applied to each randomization arm, was more conservative with respect to futility but less conservative for toxicity, reflecting its underlying decision thresholds. A utility-based framework combining efficacy and toxicity with an explicit trade-off could represent an alternative approach [35, 61, 62].

Regarding Bayesian hierarchical model, a well-known limitation is the difficulty in estimating between-arm heterogeneity when the number of arms is small [53]. While Bayesian hierarchical models are commonly applied in basket trials, the assumption of exchangeability is more likely to be violated in dose-ranging studies, which may further exhibit a limited number of randomization arms in oncology typically two to four (based on an ongoing review; PROSPERO registration CRD42025636803). This may explain the increased false positive rates observed in applying BHM to dose-ranging randomized trials. The calibrated Bayesian hierarchical model partly mitigated this issue, but at the cost of reduced power.

Bayesian logistic regression directly models dose ordering and borrowing across dose levels. However, under pharmacological assumptions consistent with a dose ranging study, forcing the regression coefficient to be strictly non negative resulted in overestimated toxicity rates, notably at the highest dose level, and therefore conservative decisions with regards to toxicity at the end of the trial. Consistently, this approach might be appropriate in dose-optimization studies aimed at identifying the minimum effective dose (pre-/post-approval), particularly when higher doses are expected to be more toxic. However, the assumption of log-linearity may be violated, for instance in the presence of plateau-shaped dose–response relationships or even inverted U-shaped relationships, as commonly observed with immunotherapies, resulting in poor performances of the approach as illustrated in our simulations with non monotonic dose-response relationships. Alternative dose-response models allowing relaxing this assumption, such as one-parameter power models with skeleton approaches as in the dose-finding Continual Reassessment Method, were found to inflate false positive rates when applied to a phase II objective in scenarios close to Ibrutinib dose-ranging use case (see Additional file 1).

Regarding prior distributions, cbhmBOP appeared more robust to prior specification than hBOP and log1BOP (see Additional file 1).

In this work, we compared several approaches for computing posterior distributions and applied them alongside the BOP2 stopping rules for futility and toxicity. Future research could compare these methods with alternative frequentist or Bayesian designs. Moreover, stopping rules, which were here defined by grid search and simulations using the framework of BOP2 design with conjugate Dirichlet-multinomial Bayesian models for efficacy and toxicity, could be calibrated specifically for each method through extensive simulations, toward optimized operating characteristics. Although BOP2 accounts for both efficacy and toxicity through multinomial modeling and allows incorporation of their correlation at the design stage, control of the type I error rate is ensured only under the global null hypothesis, that is, when all arms are both futile and toxic. Consequently, arm-specific type I error rates may exceed nominal levels in scenarios with only futile or only toxic treatments, as observed in our simulations. This limitation could be addressed through stricter FWER control or through calibration procedures that explicitly account for multiple null hypotheses [38, 40].

## Conclusion

In the setting of randomized dose-ranging studies with group-sequential designs, borrowing across arms to inform the analyses of efficacy and toxicity endpoints led to increased false positive rates in recommending arms as efficacious and safe. Bayesian optimal designs without information sharing, such as BOP2 design, adapted for randomized studies provide a robust approach, in most cases. Nevertheless, dose optimization hypotheses may further motivate the choice of more specific methods, depending on pharmacological characteristics of the treatment: in the case of strictly monotone dose-response curves, logistic regression method may prove to be more powerful. Most of all, following the recent proposals of guidance on the use of Bayesian methods in clinical trials from the FDA and the EMA [63–67], it is also critical to calibrate designs characteristics in detailed simulations; all information necessary in planning and analyzing a trial should be transparently reported to ensure reproducibility.

## Supplementary Information

Additional file 1: Additional information and results.

## Data Availability

All codes to replicate the results are provided at https://github.com/GuillaumeMulier/PrioBorrow.

## References

[1] Rubinstein L. Phase II design: history and evolution. Chin Clin Oncol. 2014;3(4):48. 25841529 10.3978/j.issn.2304-3865.2014.02.02

[2] Qi T, Dunlap T, Cao Y. Embracing project optimus: can we leverage evolutionary theory to optimize dosing in oncology? Pharm Res. 2022;39(12):3259–65. 36056271 10.1007/s11095-022-03380-1 PMC9789176

[3] Ratain MJ, Tannock IF, Lichter AS. Dose optimization of sotorasib: is the US Food and Drug Administration sending a message? J Clin Oncol. 2021;39(31):3423–6. 34543056 10.1200/JCO.21.01371

[4] Grayling MJ, Dimairo M, Mander AP, Jaki TF. A review of perspectives on the use of randomization in phase II oncology trials. JNCI J Natl Cancer Inst. 2019;111(12):1255–62. 31218346 10.1093/jnci/djz126 PMC6910171

[5] Liu B, Zhou H, Tan L, Siu KTH, Guan XY. Exploring treatment options in cancer: tumor treatment strategies. Signal Transduct Target Ther. 2024;9(1):175. 39013849 10.1038/s41392-024-01856-7 PMC11252281

[6] Hoos A. Development of immuno-oncology drugs–from CTLA4 to PD1 to the next generations. Nat Rev Drug Discovery. 2016;15(4):235–47. 26965203 10.1038/nrd.2015.35

[7] Ivanova A, Paul B, Marchenko O, Song G, Patel N, Moschos SJ. Nine-year change in statistical design, profile, and success rates of phase II oncology trials. J Biopharm Stat. 2016;26(1):141–9. 26368744 10.1080/10543406.2015.1092030 PMC5995106

[8] Wong CH, Siah KW, Lo AW. Estimation of clinical trial success rates and related parameters. Biostatistics. 2019;20(2):273–86. 29394327 10.1093/biostatistics/kxx069 PMC6409418

[9] Fourie Zirkelbach J, Shah M, Vallejo J, Cheng J, Ayyoub A, Liu J, et al. Improving dose-optimization processes used in oncology drug development to minimize toxicity and maximize benefit to patients. J Clin Oncol. 2022;40(30):3489–500. 36095296 10.1200/JCO.22.00371

[10] Roda D, Jimenez B, Banerji U. Are doses and schedules of small-molecule targeted anticancer drugs recommended by phase I studies realistic? Clin Cancer Res. 2016;22(9):2127–32. 26581244 10.1158/1078-0432.CCR-15-1855

[11] Shah M, Rahman A, Theoret MR, Pazdur R. The drug-dosing conundrum in oncology–when less is more. N Engl J Med. 2021;385(16):1445–7. 34623789 10.1056/NEJMp2109826

[12] Serritella AV, Strohbehn GW, Goldstein DA, Lichter AS, Ratain MJ. Interventional pharmacoeconomics: a novel mechanism for unlocking value. Clin Pharmacol Ther. 2020;108(3):487–93. 32298471 10.1002/cpt.1853

[13] Heiss B, Pan L, Akalu A, Vallejo J, Cheng J, Balcazar P, et al.. Dosage optimization in drug development: An FDA Project Optimus analysis of postmarketing requirements issued to repair the cracks. American Society of Clinical Oncology; 2023.

[14] Singh H, Vellanki PJ, Pazdur R. The Retrofit: Lessons From Sotorasib’s Dosing Conundrum. J Clin Oncol Off J Am Soc Clin Oncol. 2024;43(3):248–50. 10.1200/JCO.24.00310 39374478

[15] Ratain MJ. Redefining the primary objective of phase I oncology trials. Nat Rev Clin Oncol. 2014;11(9):503–4. 25091610 10.1038/nrclinonc.2014.135

[16] FDA oncology center of excellence, center for drug evaluation and research, center for biologics evaluation and research. Optimizing the dosage of human prescription drugs and biological products for the treatment of oncologic diseases guidance for industry. 2024.https://www.fda.gov/regulatory-information/search-fda-guidance-documents/optimizing-dosage-human-prescription-drugs-and-biological-products-treatment-oncologic-diseases. Accessed 29 May 2025.

[17] Gao W, Liu J, Shtylla B, Venkatakrishnan K, Yin D, Shah M, et al. Realizing the promise of Project Optimus: Challenges and emerging opportunities for dose optimization in oncology drug development. CPT Pharmacometrics Syst Pharmacol. 2024;13(5):691–709. 10.1002/psp4.13079. 37969061 10.1002/psp4.13079 PMC11098159

[18] FDA. Project Optimus. 2024. https://www.fda.gov/about-fda/oncology-center-excellence/project-optimus. Accessed 30 Dec 2025.

[19] Korn EL, Moscow JA, Freidlin B. Dose optimization during drug development: whether and when to optimize. JNCI J Natl Cancer Inst. 2023;115(5):492–7. 36534891 10.1093/jnci/djac232 PMC10165487

[20] de Claro RA, McGinn KM, Verdun N, Lee SL, Chiu HJ, Saber H, et al. FDA approval: ibrutinib for patients with previously treated mantle cell lymphoma and previously treated chronic lymphocytic leukemia. Clin Cancer Res. 2015;21(16):3586–90. 26275952 10.1158/1078-0432.CCR-14-2225

[21] Advani RH, Buggy JJ, Sharman JP, Smith SM, Boyd TE, Grant B, et al. Bruton tyrosine kinase inhibitor ibrutinib (PCI-32765) has significant activity in patients with relapsed/refractory B-cell malignancies. J Clin Oncol. 2013;31(1):88–94. 23045577 10.1200/JCO.2012.42.7906 PMC5505166

[22] Byrd JC, Furman RR, Coutre SE, Flinn IW, Burger JA, Blum KA, et al. Targeting BTK with ibrutinib in relapsed chronic lymphocytic leukemia. N Engl J Med. 2013;369(1):32–42. 23782158 10.1056/NEJMoa1215637 PMC3772525

[23] Wang ML, Rule S, Martin P, Goy A, Auer R, Kahl BS, et al. Targeting BTK with ibrutinib in relapsed or refractory mantle-cell lymphoma. N Engl J Med. 2013;369(6):507–16. 23782157 10.1056/NEJMoa1306220 PMC4513941

[24] Treon SP, Tripsas CK, Meid K, Warren D, Varma G, Green R, et al. Ibrutinib in previously treated Waldenström’s macroglobulinemia. N Engl J Med. 2015;372(15):1430–40. 25853747 10.1056/NEJMoa1501548

[25] Burger JA, Tedeschi A, Barr PM, Robak T, Owen C, Ghia P, et al. Ibrutinib as initial therapy for patients with chronic lymphocytic leukemia. N Engl J Med. 2015;373(25):2425–37. 26639149 10.1056/NEJMoa1509388 PMC4722809

[26] Shanafelt TD, Wang XV, Kay NE, Hanson CA, O’Brien S, Barrientos J, et al. Ibrutinib-rituximab or chemoimmunotherapy for chronic lymphocytic leukemia. N Engl J Med. 2019;381(5):432–43. 31365801 10.1056/NEJMoa1817073 PMC6908306

[27] Hou JZ, Ryan K, Du S, Fang B, Marks S, Page R, et al. Real-world ibrutinib dose reductions, holds and discontinuations in chronic lymphocytic leukemia. Future Oncol. 2021;17(35):4959–69. 34783255 10.2217/fon-2021-0964

[28] Chen LS, Bose P, Cruz ND, Jiang Y, Wu Q, Thompson PA, et al. A pilot study of lower doses of ibrutinib in patients with chronic lymphocytic leukemia. Blood J Am Soc Hematol. 2018;132(21):2249–59. 10.1182/blood-2018-06-860593 PMC6251009 30254130

[29] Yan F, Thall P, Lu K, Gilbert M, Yuan Y. Phase I-II clinical trial design: a state-of-the-art paradigm for dose finding. Ann Oncol. 2018;29(3):694–9. 29267863 10.1093/annonc/mdx795 PMC5888967

[30] Lin R, Zhou Y, Yan F, Li D, Yuan Y. BOIN12: Bayesian optimal interval phase I/II trial design for utility-based dose finding in immunotherapy and targeted therapies. JCO Precis Oncol. 2020;4:1393–402. 10.1200/PO.20.00257 PMC7713525 33283133

[31] Guo B, Yuan Y. DROID: dose-ranging approach to optimizing dose in oncology drug development. Biometrics. 2023;79(4):2907–19. 36807110 10.1111/biom.13840 PMC11713780

[32] Zhang J, Chen X, Li B, Yan F. A comparative study of adaptive trial designs for dose optimization. Pharm Stat. 2023;22(5):797–814. 37156731 10.1002/pst.2306

[33] Yuan Y, Nguyen HQ, Thall PF. Bayesian designs for phase I-II clinical trials. New York: Chapman & Hall/CRC; 2016.

[34] Jaki T, Burdon A, Chen X, Mozgunov P, Zheng H, Baird R. Early phase clinical trials in oncology: Realising the potential of seamless designs. Eur J Cancer. 2023;189:112916. 37301716 10.1016/j.ejca.2023.05.005 PMC7614750

[35] Yuan Y, Zhou H, Liu S. Statistical and practical considerations in planning and conduct of dose-optimization trials. Clin Trials. 2024;21(3):273–86. 38243399 10.1177/17407745231207085 PMC11134987

[36] Araujo D, Greystoke A, Bates S, Bayle A, Calvo E, Castelo-Branco L, et al. Oncology phase I trial design and conduct: time for a change-MDICT Guidelines 2022. Ann Oncol. 2023;34(1):48–60. 36182023 10.1016/j.annonc.2022.09.158

[37] Dragalin V, Bornkamp B, Bretz F, Miller F, Padmanabhan SK, Patel N, et al. A simulation study to compare new adaptive dose-ranging designs. Stat Biopharm Res. 2010;2(4):487–512.

[38] Chen K, Zhou H, Lee JJ, Yuan Y. BOP2-TE: Bayesian optimal phase 2 design for jointly monitoring efficacy and toxicity with application to dose optimization. J Biopharm Stat. 2026;36(1):43–58. 10.1080/10543406.2024.2429481 PMC12102293 39582234

[39] Neuenschwander B, Wandel S, Roychoudhury S, Bailey S. Robust exchangeability designs for early phase clinical trials with multiple strata. Pharm Stat. 2016;15(2):123–34. 26685103 10.1002/pst.1730

[40] Daniells L, Mozgunov P, Barnett H, Bedding A, Jaki T. How to add baskets to an ongoing basket trial with information borrowing. Stat Methods Med Res. 2025;34(4):717–34. 10.1177/09622802251316961 PMC12075893 40111817

[41] Berry SM, Broglio KR, Groshen S, Berry DA. Bayesian hierarchical modeling of patient subpopulations: efficient designs of phase II oncology clinical trials. Clin Trials. 2013;10(5):720–34. 23983156 10.1177/1740774513497539 PMC4319656

[42] Chu Y, Yuan Y. A Bayesian basket trial design using a calibrated Bayesian hierarchical model. Clin Trials. 2018;15(2):149–58. 29499621 10.1177/1740774518755122 PMC5891374

[43] Zhang J, Zhou H, Wages NA, Guo Z, Liu F, Jemielita T, et al. TODO: A Triple-Outcome Double-Criterion Optimal Design for Dose Monitoring-and-Optimization in Multi-Dose Randomized Trials. Stat Med. 2025;44(10–12):e70090. 40390185 10.1002/sim.70090 PMC12089520

[44] Gajewski BJ, Meinzer C, Berry SM, Rockswold GL, Barsan WG, Korley FK, et al. Bayesian hierarchical EMAX model for dose-response in early phase efficacy clinical trials. Stat Med. 2019;38(17):3123–38. 31070807 10.1002/sim.8167 PMC6606375

[45] Huang X, Gajewski BJ. Comparison of hierarchical EMAX and NDLM models in dose-response for early phase clinical trials. BMC Med Res Methodol. 2020;20(1):194. 32690004 10.1186/s12874-020-01071-2 PMC7370408

[46] Yada S, Hamada C. Application of Bayesian hierarchical models for phase I/II clinical trials in oncology. Pharm Stat. 2017;16(2):114–21. 27892650 10.1002/pst.1793

[47] Jiménez JL, Zheng H. A Bayesian adaptive design for dual-agent phase I-II oncology trials integrating efficacy data across stages. Biom J. 2023;65(7):2200288. 37199700 10.1002/bimj.202200288 PMC10952513

[48] Jiang Z, Mi G, Lin J, Lorenzato C, Ji Y. A Multi-Arm Two-Stage (MATS) design for proof-of-concept and dose optimization in early-phase oncology trials. Contemp Clin Trials. 2023;132:107278. 37419308 10.1016/j.cct.2023.107278

[49] Mulier G, Chevret S, Lin R, Biard L. Bayesian optimal designs for multi-arm multi-stage phase II randomized clinical trials with multiple endpoints. Stat Biopharm Res. 2024;16(3):315–25. 10.1080/19466315.2024.2344543 PMC11412438 39301054

[50] Zhou H, Lee JJ, Yuan Y. BOP2: Bayesian optimal design for phase II clinical trials with simple and complex endpoints. Stat Med. 2017;36(21):3302–14. 28589563 10.1002/sim.7338

[51] Thall PF, Simon R, Ellenberg SS. A two-stage design for choosing among several experimental treatments and a control in clinical trials. Biometrics. 1989;45(2):537–47. 2765637

[52] Chen MH, Ibrahim JG. Power prior distributions for regression models. Stat Sci. 2000;15(1):46–60.

[53] Spiegelhalter DJ, Abrams KR, Myles JP. Bayesian approaches to clinical trials and health-care evaluation. Wiley; 2004.

[54] Neuenschwander B, Branson M, Gsponer T. Critical aspects of the Bayesian approach to phase I cancer trials. Stat Med. 2008;27(13):2420–39. 18344187 10.1002/sim.3230

[55] Simon R. Optimal two-stage designs for phase II clinical trials. Control Clin Trials. 1989;10(1):1–10. 2702835 10.1016/0197-2456(89)90015-9

[56] Ivanova A, Qaqish BF, Schell MJ. Continuous toxicity monitoring in phase II trials in oncology. Biometrics. 2005;61(2):540–5. 16011702 10.1111/j.1541-0420.2005.00311.x

[57] Thall PF, Simon RM, Estey EH. Bayesian sequential monitoring designs for single-arm clinical trials with multiple outcomes. Stat Med. 1995;14(4):357–79. 7746977 10.1002/sim.4780140404

[58] Schmidli H, Gsteiger S, Roychoudhury S, O’Hagan A, Spiegelhalter D, Neuenschwander B. Robust meta-analytic-predictive priors in clinical trials with historical control information. Biometrics. 2014;70(4):1023–32. 25355546 10.1111/biom.12242

[59] Zhao Y, Yang B, Lee JJ, Wang L, Yuan Y. Bayesian optimal phase II design for randomized clinical trials. Stat Biopharm Res. 2022;14(4):423–32.

[60] Viele K, Berry S, Neuenschwander B, Amzal B, Chen F, Enas N, et al. Use of historical control data for assessing treatment effects in clinical trials. Pharm Stat. 2014;13(1):41–54. 23913901 10.1002/pst.1589 PMC3951812

[61] Thall PF, Cook JD. Dose-finding based on efficacy-toxicity trade-offs. Biometrics. 2004;60(3):684–93. 15339291 10.1111/j.0006-341X.2004.00218.x

[62] Zhou Y, Lee JJ, Yuan Y. A utility-based Bayesian optimal interval (U-BOIN) phase I/II design to identify the optimal biological dose for targeted and immune therapies. Stat Med. 2019;38(28):S5299–316. 10.1002/sim.8361 PMC7927960 31621952

[63] FDA. Draft guidance, Use of Bayesian Methodology in Clinical Trials of Drug and Biological Products, Guidance for Industry. 2026. https://www.fda.gov/media/190505/download. Accessed 24 Mar 2026.

[64] EMA. Concept Paper for the Development of a Reflection Paper 4 on the use of Bayesian methods in clinical development. 2026. https://www.ema.europa.eu/en/documents/scientific-guideline/concept-paper-development-reflection-paper-use-bayesian-methods-clinical-development_en.pdf. Accessed 24 Mar 2026.

[65] Evans SR, Fleming TR, Janes H, Dodd LE. Reflections on FDA draft guidance on Bayesian methods in trials—protecting scientific integrity and evidentiary standards. JAMA. 10.1001/jama.2026.4175. Published online 23 Mar 2026. 10.1001/jama.2026.4175 PMC13508263 41870422

[66] Gelman A, van Zwet E, Więcek W. FDA draft guidance for the use of Bayesian methods in clinical trials. JAMA. 10.1001/jama.2026.4178. Published online 23 Mar 2026. 10.1001/jama.2026.4178 41870890

[67] Lee JJ, Harrell Jr FE, LaVange LM, Spiegelhalter DJ. Embracing Bayesian methods in clinical trials: FDA’s long-awaited draft guidance. JAMA. 10.1001/jama.2026.4179. Published online 2026. 10.1001/jama.2026.4179 41870893

