# Supplementary material for: Information borrowing in phase II randomized dose-ranging clinical trials in oncology
Source: BMC Med Res Methodol. 2026 May 27;26:164. doi: 10.1186/s12874-026-02883-4 (PMC13397689; doi:10.1186/s12874-026-02883-4)
Supplement: Supplementary file 1 — Additional file 1: Additional information and results. [file 12874_2026_2883_MOESM1_ESM.pdf]

Additional file 1 — Additional information and results

1 Scenarios of simulation

Table S1: Parameters of the multinomial distribution used to simulated each scenario (presented as $(\Pr(\text{Eff} \cap \text{Tox}), \Pr(\text{Eff} \cap \text{NoTox}), \Pr(\text{noEff} \cap \text{Tox}), \Pr(\text{NoEff} \cap \text{NoTox})))$

Scenario	Arm 1	Arm 2	Arm 3
Sc1	(0.15;0.15;0.25;0.45)	(0.15;0.15;0.25;0.45)	(0.15;0.15;0.25;0.45)
Sc2	(0.13;0.12;0.27;0.48)	(0.15;0.13;0.27;0.45)	(0.16;0.14;0.29;0.41)
Sc3	(0.20;0.30;0.10;0.40)	(0.20;0.30;0.10;0.40)	(0.20;0.30;0.10;0.40)
Sc4	(0.15;0.35;0.10;0.40)	(0.17;0.35;0.11;0.37)	(0.19;0.36;0.11;0.34)
Sc5	(0.11;0.19;0.17;0.53)	(0.20;0.30;0.10;0.40)	(0.25;0.30;0.15;0.30)
Sc6	(0.14;0.26;0.14;0.46)	(0.20;0.30;0.10;0.40)	(0.25;0.30;0.15;0.30)
Sc7	(0.18;0.32;0.12;0.38)	(0.22;0.28;0.15;0.35)	(0.23;0.27;0.17;0.33)
Sc8	(0.12;0.18;0.18;0.52)	(0.17;0.23;0.18;0.42)	(0.23;0.27;0.17;0.33)
Sc9	(0.12;0.18;0.18;0.52)	(0.23;0.27;0.17;0.33)	(0.17;0.23;0.18;0.42)
ScI1	(0.10;0.20;0.15;0.55)	(0.20;0.30;0.10;0.40)	(0.19;0.36;0.11;0.34)
ScI2	(0.10;0.20;0.15;0.55)	(0.19;0.36;0.11;0.34)	(0.19;0.36;0.11;0.34)
ScI3	(0.15;0.35;0.10;0.40)	(0.18;0.34;0.12;0.36)	(0.25;0.30;0.15;0.30)

2 Calibration of designs

Calibration of multi-arm BOP2 design

As described in the main manuscript, the decisions rules for the mBOP design in each arm k are defined as follows :

1. Stop arm k for futility if $Pr(p_{k,\text{eff}} \leq \phi_{\text{eff}} \mid D_{n_j,k}) > C_{n_j}$

2. Stop arm k for toxicity if $Pr(p_{k,\text{tox}} > \phi_{\text{tox}} \mid D_{n_j,k}) > C_{n_j}$

where $C_{n_j} = 1 - \lambda \left(\frac{n_{j,k}}{N}\right)^{\gamma}$, with $n_{j,k}$ the number of patients accrued at the analysis j in each arm k, N the maximum number of patients per arm, and λ and γ two tuning hyperparameters.

To optimize λ and γ, we simulated 10,000 trials under the global null hypothesis and 10,000 trials under the Least Favourable Configuration. We then iterated over pairs (λ, γ) and selected the combination yielding the highest power (defined as the proportion of trials correctly identifying the efficacious and non-toxic arm under the Least Favourable Configuration), while maintaining the family-wise error rate (FWER), defined as the proportion of trials declaring at least one promising arm under the global null hypothesis, below the prespecified threshold.

In all cases, the interim analysis was performed when half of the planned sample size per arm had been accrued.

The resulting calibrated thresholds are the following :

1. 3 arms: $N = 58$, $\lambda = 0.76$, $\gamma = 1.31$, FWER = 9.13%, and arm-level power = 80.64%;
2. 4 arms: $N = 70$, $\lambda = 0.775$, $\gamma = 1.05$, FWER = 9.90%, and arm-level power = 80.75%;
3. 5 arms: $N = 70$, $\lambda = 0.79$, $\gamma = 1.05$, FWER = 9.84%, and arm-level power = 79.97%.

Calibration of Simon+TM procedure

Simon's two-stage design monitors efficacy only. Therefore, we added a toxicity monitoring rule (TM) based on the posterior probability of toxicity. In order to control the FWER globally, we targeted a false positive rate of $\frac{0.1}{K}$ in each arm (Bonferroni correction) and then optimized both components of the design to achieve the target α level under the assumption of independence. For instance, with 3 arms, the target FWER is $\frac{0.1}{3} = 0.033$. By combining a Simon's two-stage design with $\alpha = 0.1$ and a posterior probability–based toxicity monitoring with $\alpha = 0.33$, we expect to achieve the desired global FWER under independence assumption.

Simon's two-stage design

Simon's two-stage design requires specification of the type I and type II error rates, the unacceptable response rate under the null hypothesis, and the desirable response rate under the alternative hypothesis. Using binomial enumeration under the null and alternative hypotheses, the type I error and power are computed for all possible stopping rules. The optimal design is defined as the one satisfying the type I error and power constraints while minimizing the expected sample size under the null hypothesis.

Because the BOP2 design was calibrated with fixed sample sizes at interim and final analyses, we constrained Simon's two-stage design to use the same sample sizes to allow fair comparison. We then identified the optimal Simon design under these constraints, which corresponded to 10% type I error risk and 80% power.

The resulting designs were as follows (main analyses with 3 arms, sensitivity analyses with 4 and 5 arms):

1. 3 arms: $\alpha = 8.3\%$, power = 86.6%, stop for futility at first analysis if there are less than 12 responses among the first 29 patients and at final analysis the treatment is deemed efficacious if there are more than 20 responses among the 58 patients;
2. 4 arms: $\alpha = 6.1\%$, power = 84.5%, stop for futility at first analysis if there are less than 15 responses among the first 35 patients and at final analysis the treatment is deemed efficacious if there are more than 23 responses among the 70 patients;
3. 5 arms: $\alpha = 6.1\%$, power = 84.5%, stop for futility at first analysis if there are less than 15 responses among the first 35 patients and at final analysis the treatment is deemed efficacious if there are more than 23 responses among the 70 patients.

Posterior probability-based monitoring of toxicity

For toxicity monitoring, an arm k is stopped if $Pr(p_{\text{k,tox}} > \eta \mid D_{n,k}) > \tau$. The parameters η and τ were optimized through grid search, and the posterior distribution of toxicity was obtained using a conjugate beta-binomial model with a uniform prior (Beta$(1, 1)$).

We simulated 10,000 trials under the null hypothesis (toxic dose) and 10,000 trials under the alternative hypothesis (non-toxic dose). For each pair (η, τ), we computed the corresponding type I error and power, and selected the combination achieving the target type I error while maximizing power.

The resulting designs were:

1. 3 arms: $N = 58$, $\alpha = 37.7\%$, power = 85.9%, $\eta = 0.4$ and $\tau = 0.5$;
2. 4 arms: $N = 70$, $\alpha = 41.4\%$, power = 93.3%, $\eta = 0.3$ and $\tau = 0.96$;
3. 5 arms: $N = 70$, $\alpha = 31.1\%$, power = 85.3%, $\eta = 0.4$ and $\tau = 0.5$;

3 Normalized power prior analysis

The power prior used in the main manuscript is the fixed weight power prior. Here we detail the results for the normalized power prior (Carvalho LM, Ibrahim JG. On the normalized power prior. Stat Med. 2021 Oct 30;40(24):5251-5275) which estimates the parameter α_0 that controls the degree of borrowing. This estimation is made by MCMC. The models for efficacy and toxicity are the following:

$p_{j,k,\text{eff}}|D_{n_j,k} \sim \text{Beta}(\pi_{0,1} + \pi_{0,2} + \sum_{l=1;l\neq k}^{K} \alpha_{0,l,\text{eff}} x_{j,l,\text{eff}} + x_{j,k,\text{eff}},$
$\pi_{0,3} + \pi_{0,4} + \sum_{l=1;l\neq k}^{K} \alpha_{0,l,\text{eff}}(n_{j,l} - x_{j,l,\text{eff}}) + n_{j,k} - x_{j,k,\text{eff}})$
$\alpha_{0,l,\text{eff}} \sim \text{Beta}(1,1)$
$p_{j,k,\text{tox}}|D_{n_j,k} \sim \text{Beta}(\pi_{0,1} + \pi_{0,3} + \sum_{l=1;l\neq k}^{K} \alpha_{0,l,\text{tox}} x_{j,l,\text{tox}} + x_{j,k,\text{eff}},$
$\pi_{0,2} + \pi_{0,4} + \sum_{l=1;l\neq k}^{K} \alpha_{0,l,\text{tox}}(n_{j,l} - x_{j,l,\text{tox}}) + n_{j,k} - x_{j,k,\text{tox}})$
$\alpha_{0,l,\text{tox}} \sim \text{Beta}(1,1)$

Results about the 12 scenarios over 5000 simulations of 3-arm trials (as defined in the main manuscript simulation) are presented in figure S1. It is refered to as "normpowBOP" in the following.

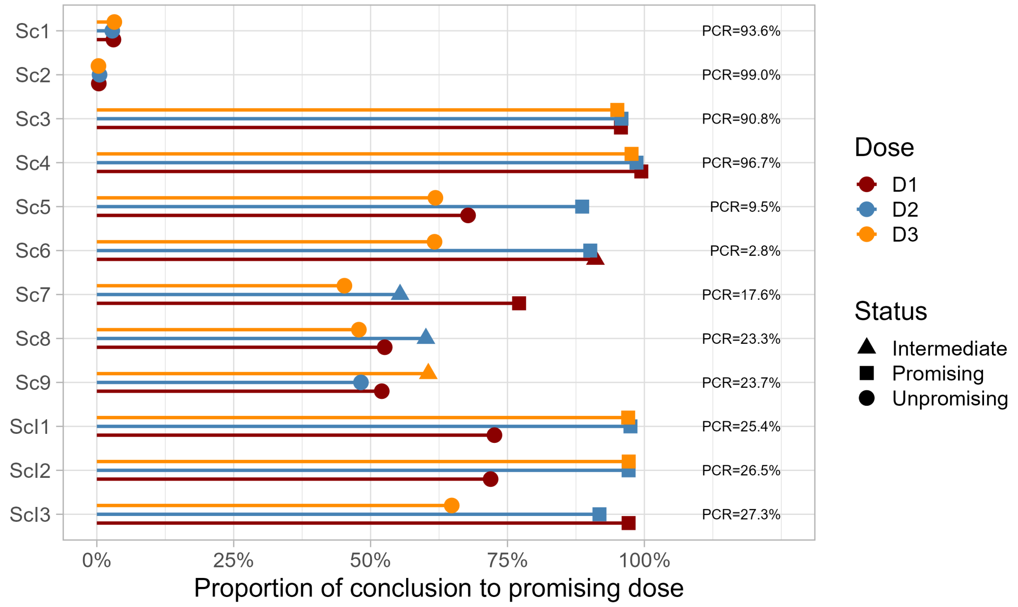

Figure S1: Proportion of conclusions in favor of a promising treatment within each arm for all scenarios for normalized power prior. The shape at the end of the bar reflect the promisingness of the dose. Doses are ordered 3-2-1 from top to bottom for each design. PCR stands for percentage of correct recommendation, that is recommending all promising doses and only them.

4 Additional results for the main analysis

Table S2: Percentage of early stoppings and mean number of patients in the 12 scenarios over 5,000 simulated trials (AP is early stoppings and MPts is mean number of patients)

		mBOP		Simon+TM		powBOP		hBOP		cbhmBOP		log1BOP		log2BOP	
Scenario	Dose	AP	MPts	AP	MPts	AP	MPts	AP	MPts	AP	MPts	AP	MPts	AP	MPts
Sc1	Dose 1	49.4%	43.7	94.8%	30.5	49.2%	43.7	50.5%	43.3	54.5%	42.2	53.7%	42.4	70.1%	37.7
	Dose 2	50.1%	43.5	94.7%	30.5	50.4%	43.4	52.2%	42.9	55.5%	41.9	55.9%	41.8	55.6%	41.9
	Dose 3	49.2%	43.7	94.7%	30.5	49.8%	43.6	50.7%	43.3	54.4%	42.2	54.3%	42.3	67.0%	38.6
Sc2	Dose 1	68.3%	38.2	98.7%	29.4	73.0%	36.8	72.9%	36.9	73.3%	36.7	72.6%	36.9	87.2%	32.7
	Dose 2	61.4%	40.2	97.2%	29.8	71.3%	37.3	70.6%	37.5	68.3%	38.2	78.4%	35.3	78.3%	35.3
	Dose 3	63.3%	39.6	97.2%	29.8	72.1%	37.1	71.4%	37.3	70.0%	37.7	69.3%	37.9	83.8%	33.7
Sc3	Dose 1	3.2%	57.1	24.3%	50.9	0.5%	57.9	1.5%	57.6	4.8%	56.6	3.2%	57.1	0.3%	57.9
	Dose 2	3.0%	57.1	24.1%	51.0	0.4%	57.9	1.2%	57.6	4.3%	56.8	0.5%	57.8	0.5%	57.8
	Dose 3	3.5%	57.0	26.9%	50.2	0.7%	57.8	1.7%	57.5	5.0%	56.5	3.9%	56.9	7.3%	55.9
Sc4	Dose 1	0.7%	57.8	17.4%	53.0	0.1%	58.0	0.3%	57.9	1.1%	57.7	0.6%	57.8	0.1%	58.0
	Dose 2	1.5%	57.6	17.3%	53.0	0.2%	57.9	0.7%	57.8	2.7%	57.2	0.2%	58.0	0.2%	58.0
	Dose 3	3.0%	57.1	17.1%	53.0	0.2%	57.9	1.4%	57.6	4.5%	56.7	3.6%	57.0	5.2%	56.5
Sc5	Dose 1	33.7%	48.2	89.0%	32.2	1.5%	57.6	14.9%	53.7	34.4%	48.0	20.3%	52.1	20.5%	52.1
	Dose 2	3.2%	57.1	25.9%	50.5	1.4%	57.6	2.4%	57.3	4.8%	56.6	2.1%	57.4	2.1%	57.4
	Dose 3	23.3%	51.2	53.2%	42.6	5.0%	56.6	14.9%	53.7	29.0%	49.6	23.6%	51.2	31.1%	49.0
Sc6	Dose 1	7.1%	55.9	53.8%	42.4	1.3%	57.6	3.1%	57.1	7.8%	55.7	4.5%	56.7	3.8%	56.9
	Dose 2	3.3%	57.0	25.9%	50.5	1.7%	57.5	2.3%	57.3	4.8%	56.6	2.1%	57.4	2.1%	57.4
	Dose 3	23.5%	51.2	53.0%	42.6	4.9%	56.6	14.7%	53.7	28.3%	49.8	23.8%	51.1	31.6%	48.8
Sc7	Dose 1	3.1%	57.1	24.5%	50.9	4.2%	56.8	4.0%	56.8	4.7%	56.6	4.0%	56.8	0.6%	57.8
	Dose 2	14.5%	53.8	46.7%	44.5	8.2%	55.6	12.1%	54.5	18.7%	52.6	7.2%	55.9	7.2%	55.9
	Dose 3	24.3%	50.9	58.3%	41.1	11.1%	54.8	18.5%	52.6	29.2%	49.5	33.3%	48.3	46.6%	44.5
Sc8	Dose 1	33.7%	48.2	89.5%	32.0	5.9%	56.3	16.8%	53.1	35.0%	47.9	32.9%	48.5	35.3%	47.8
	Dose 2	14.3%	53.8	65.2%	39.1	6.6%	56.1	10.3%	55.0	18.1%	52.7	6.6%	56.1	6.6%	56.1
	Dose 3	23.4%	51.2	58.2%	41.1	9.6%	55.2	17.6%	52.9	28.0%	49.9	29.9%	49.3	42.6%	45.6
Sc9	Dose 1	33.7%	48.2	89.5%	32.0	5.6%	56.4	16.7%	53.2	34.9%	47.9	18.3%	52.7	18.5%	52.6
	Dose 2	23.8%	51.1	58.6%	41.0	9.6%	55.2	18.0%	52.8	28.9%	49.6	7.0%	56.0	7.0%	56.0
	Dose 3	15.0%	53.6	65.7%	38.9	7.3%	55.9	11.0%	54.8	18.2%	52.7	20.9%	52.0	35.2%	47.8
ScI1	Dose 1	33.2%	48.4	88.1%	32.5	0.5%	57.8	14.2%	53.9	33.5%	48.3	20.0%	52.2	20.4%	52.1
	Dose 2	3.1%	57.1	24.1%	51.0	0.2%	57.9	1.4%	57.6	4.9%	56.6	0.2%	57.9	0.2%	57.9
	Dose 3	2.9%	57.2	16.5%	53.2	0.4%	57.9	1.1%	57.7	4.5%	56.7	3.6%	56.9	6.2%	56.2
ScI2	Dose 1	33.2%	48.4	88.1%	32.5	0.3%	57.9	14.8%	53.7	33.6%	48.3	15.5%	53.5	15.3%	53.6
	Dose 2	2.9%	57.1	17.0%	53.1	0.3%	57.9	1.3%	57.6	4.2%	56.8	0.2%	58.0	0.2%	57.9
	Dose 3	3.0%	57.1	16.6%	53.2	0.2%	57.9	1.2%	57.7	4.3%	56.8	3.7%	56.9	6.1%	56.2
ScI3	Dose 1	0.7%	57.8	17.4%	53.0	0.7%	57.8	0.6%	57.8	1.2%	57.7	0.6%	57.8	0.2%	57.9
	Dose 2	3.2%	57.1	21.5%	51.8	1.2%	57.7	2.2%	57.4	5.1%	56.5	1.2%	57.7	1.2%	57.7
	Dose 3	23.3%	51.2	52.9%	42.7	3.8%	56.9	14.5%	53.8	29.4%	49.5	25.2%	50.7	30.6%	49.1

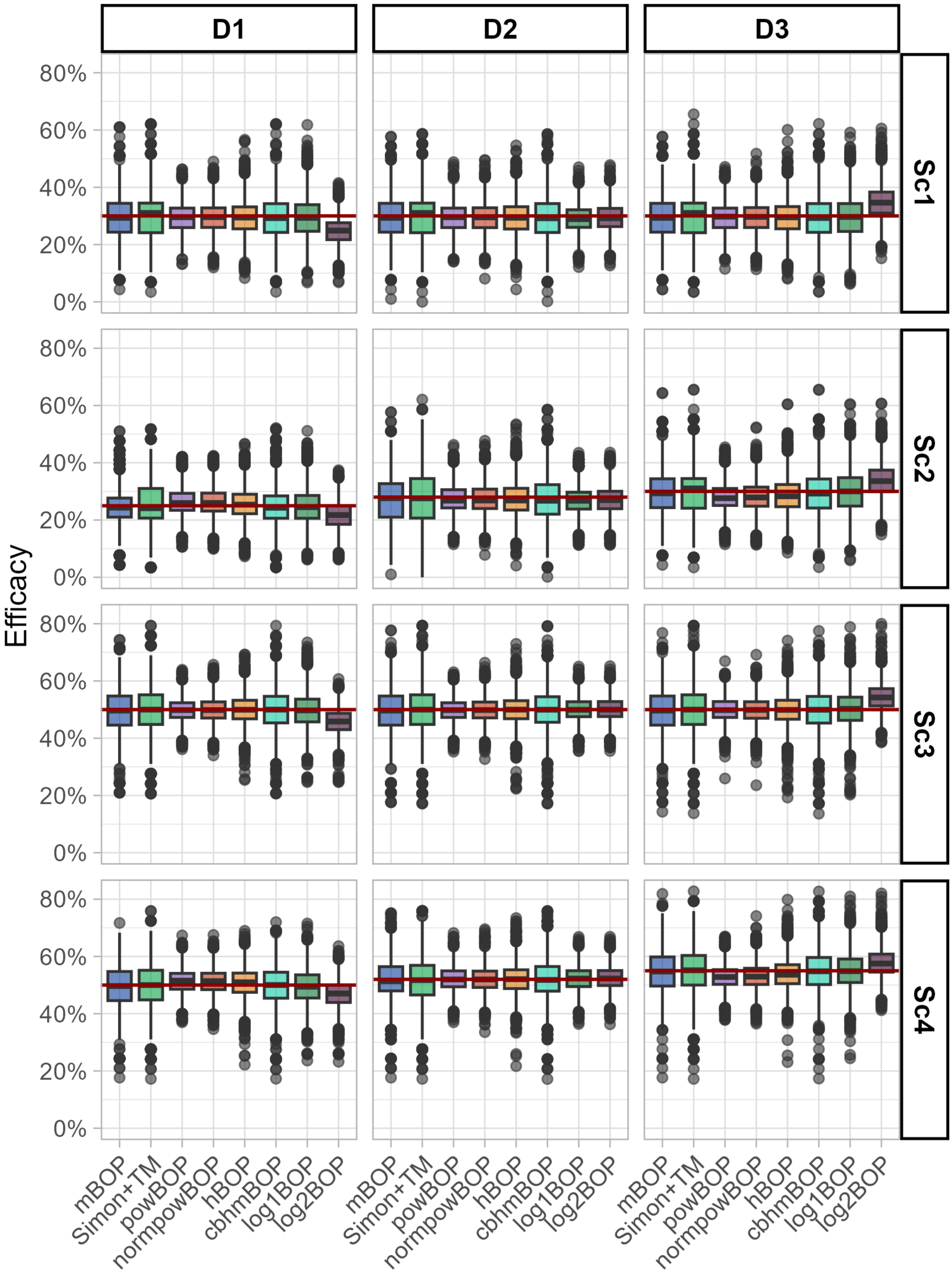

Figure S2: Efficacy estimates for each simulated trials in scenarios 1, 2, 3 and 4. The red line is the true efficacy rate.

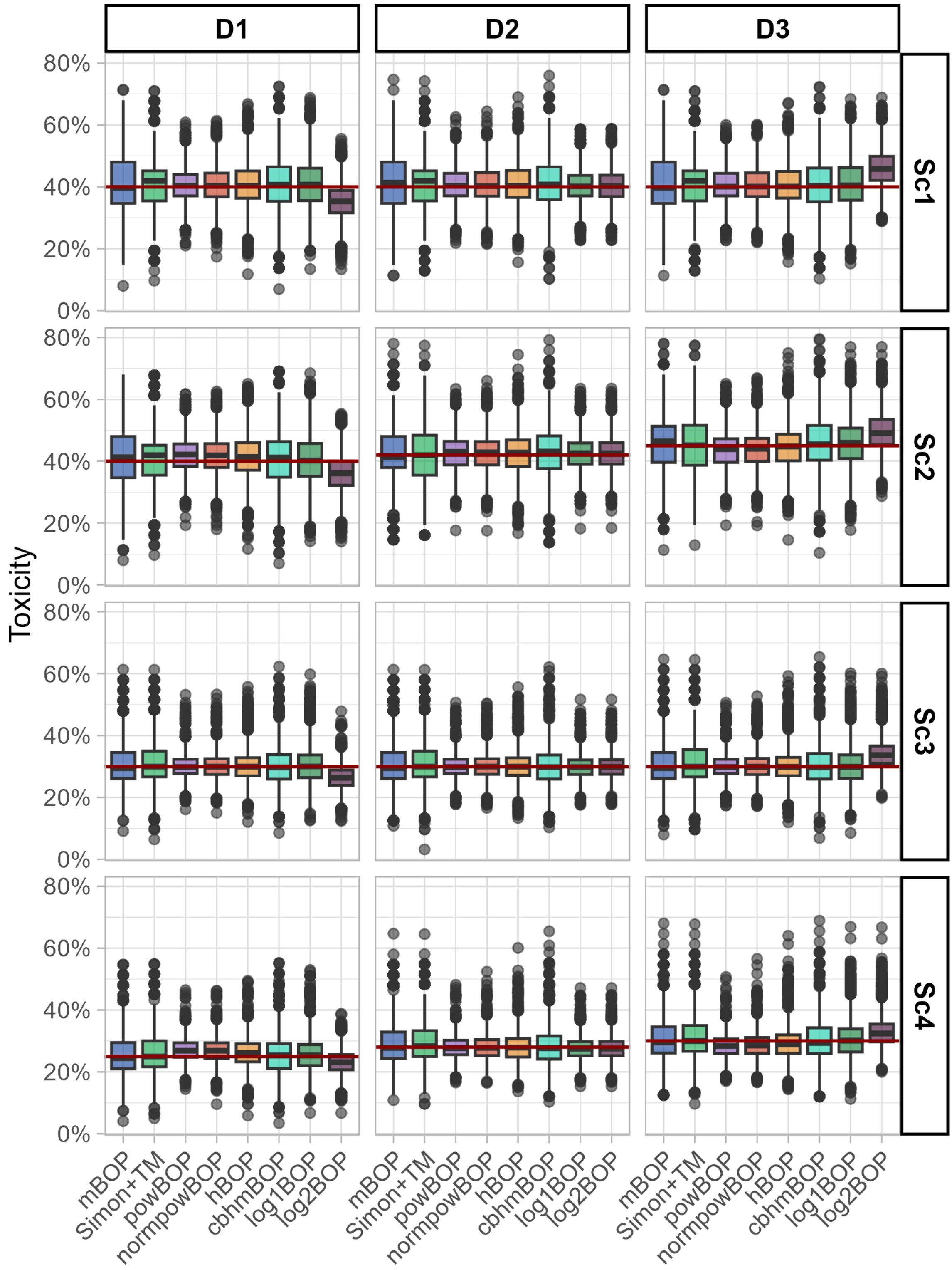

Figure S3: Toxicity estimates for each simulated trials in scenarios 1, 2, 3 and 4. The red line is the true toxicity rate.

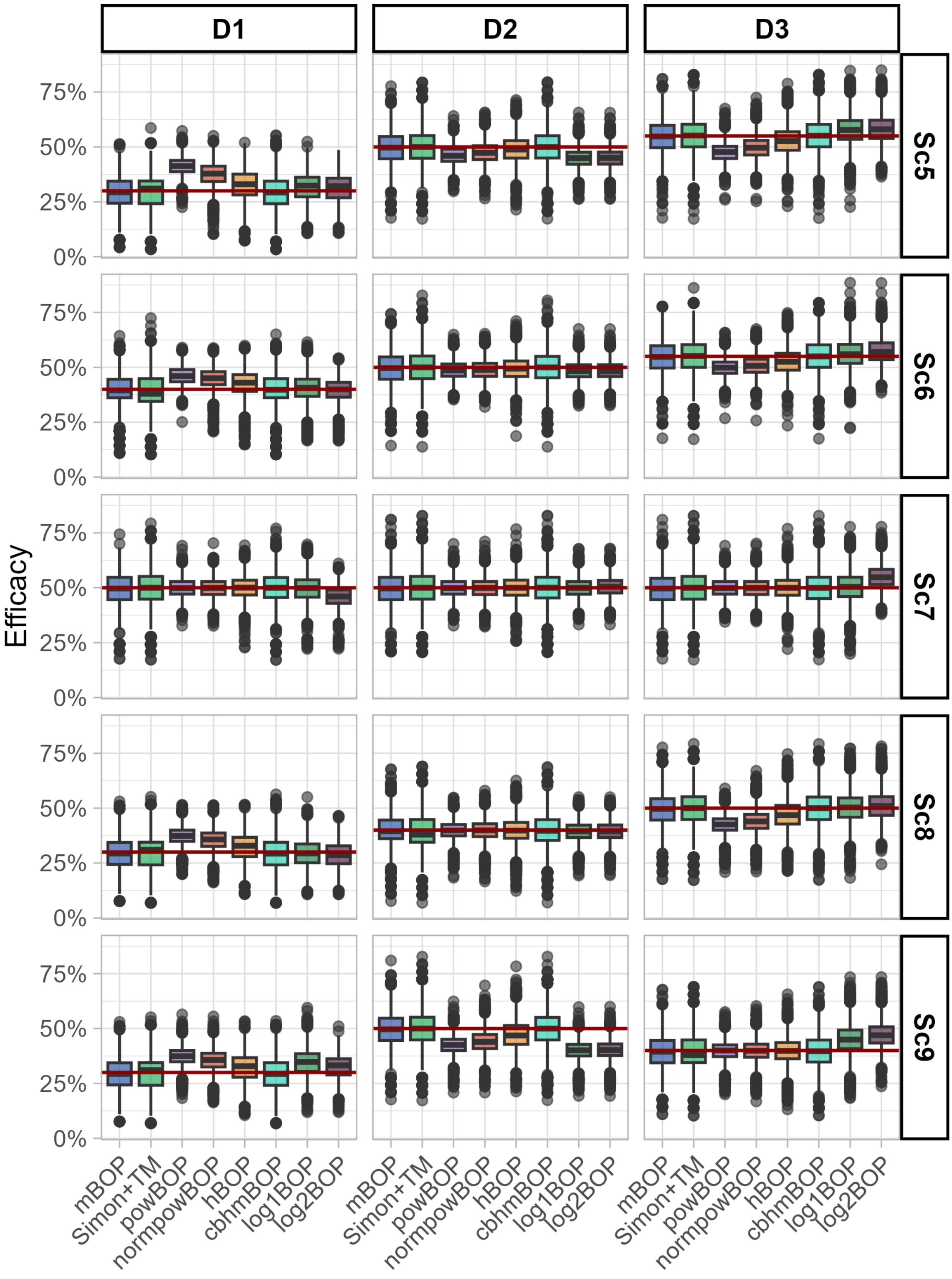

Figure S4: Efficacy estimates for each simulated trials in scenarios 5, 6, 7, 8 and 9. The red line is the true efficacy rate.

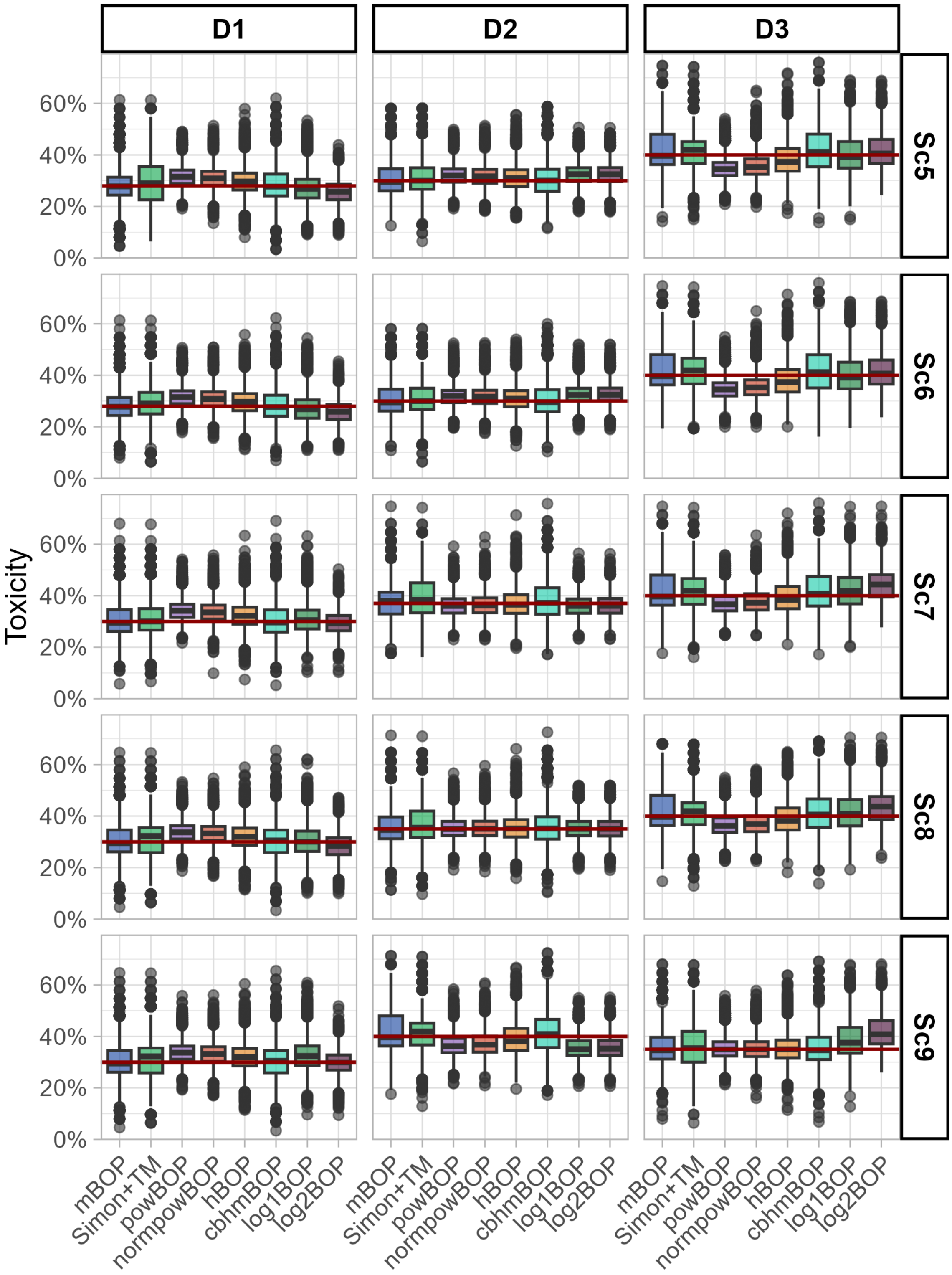

Figure S5: Toxicity estimates for each simulated trials in scenarios 5, 6, 7, 8 and 9. The red line is the true toxicity rate.

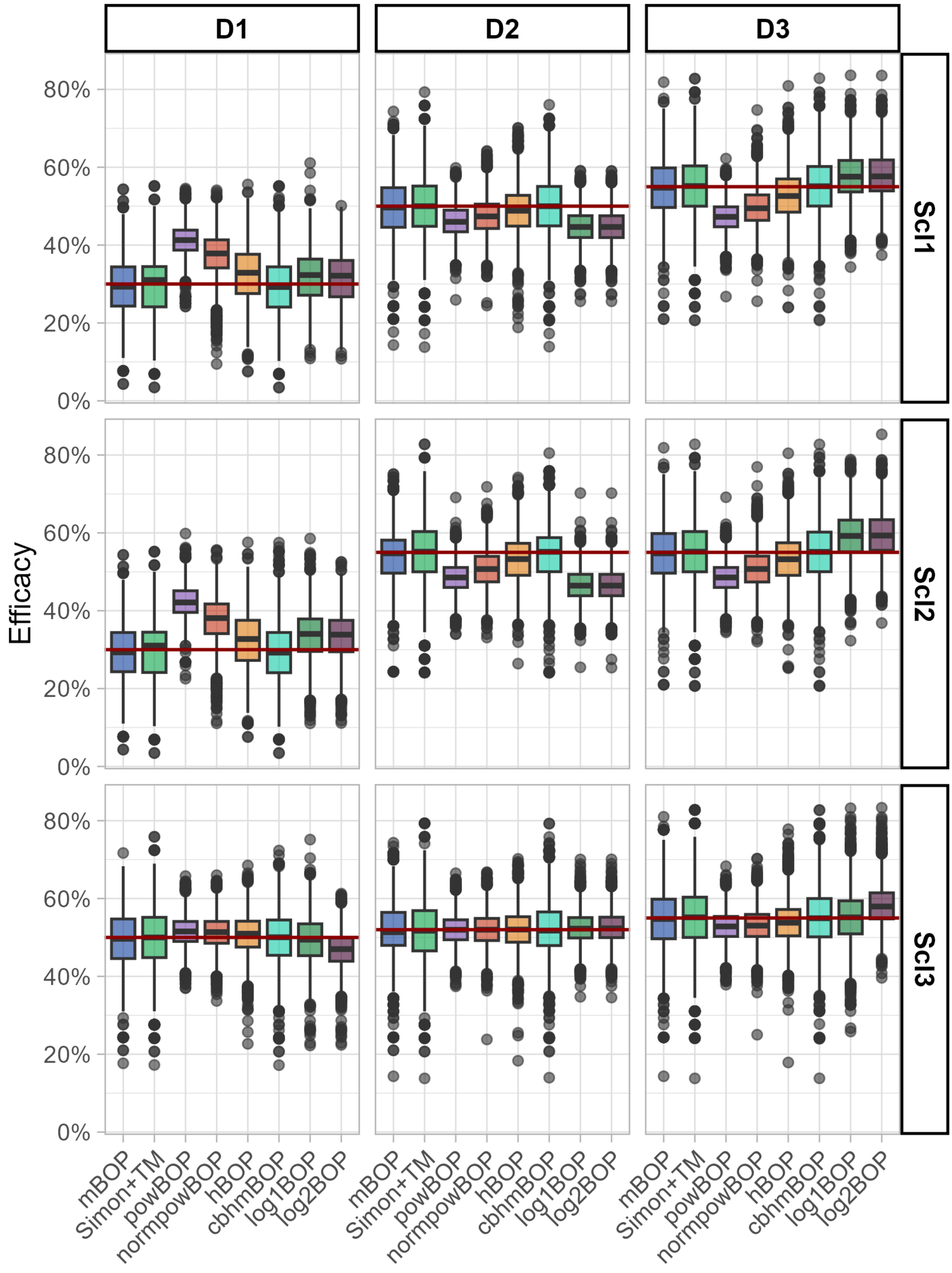

Figure S6: Efficacy estimates for each simulated trials in scenarios I1 and I3. The red line is the true efficacy rate.

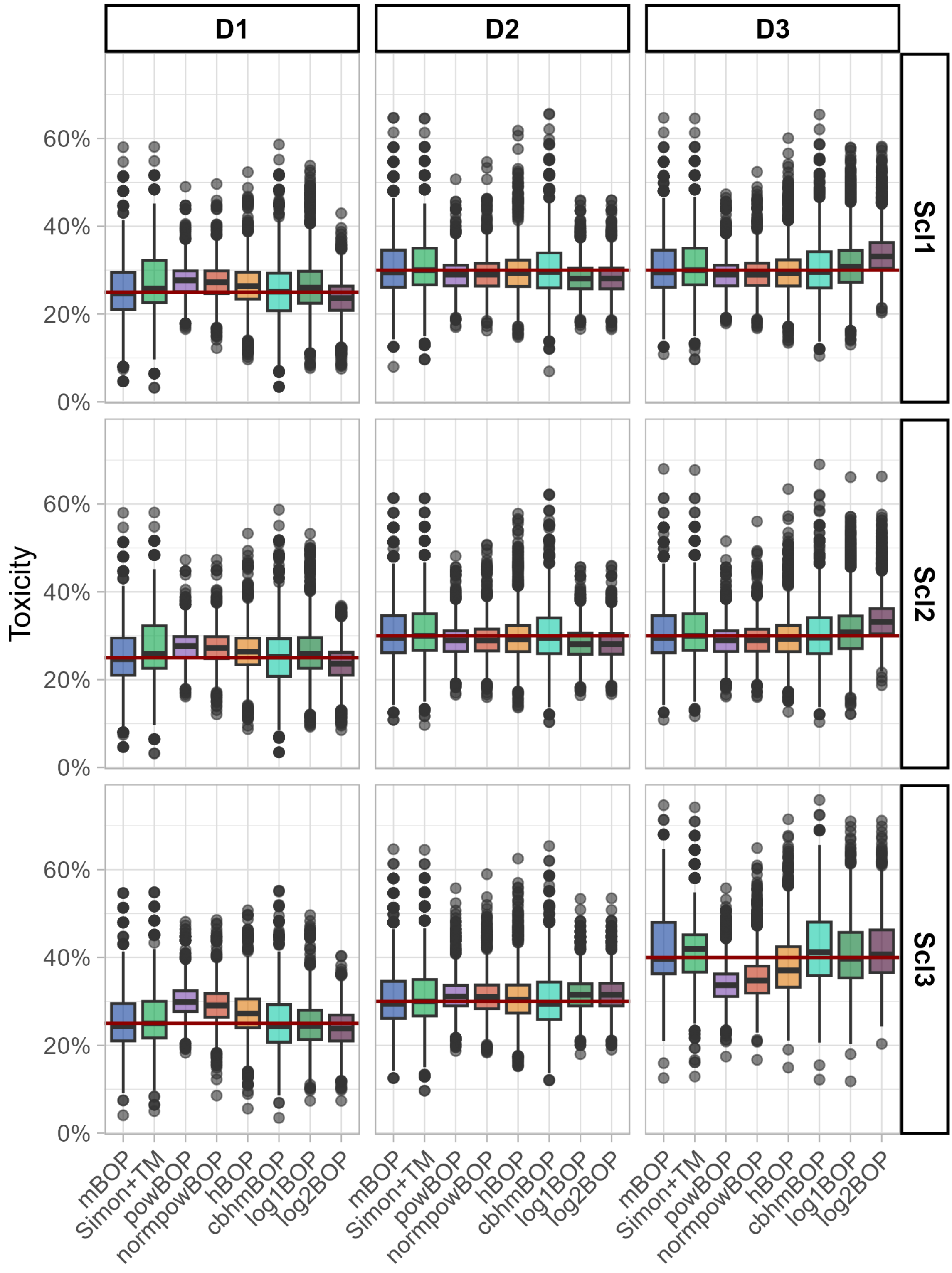

Figure S7: Toxicity estimates for each simulated trials in scenarios I1 and I3. The red line is the true toxicity rate.

5 Sensitivity analysis: more than 3 arms

The main analysis used 3 arms, with regards to the Ibrutinib use case and potential dose levels. We assessed the impact of increasing the number of randomized arms, *i.e.*, 4 or 5 arms. Including more than 5 arms in a dose-ranging study is likely unrealistic in the context of a trial conducted after a phase I study in oncology that would have ruled out toxic doses. We restricted the analysis to the most clinically relevant scenarios, namely scenarios 2, 4, I1, and I3. Simulation settings for 4 and 5 arms were as follows:

- inefficacy/toxicity hypothesis: $\pi_{\text{eff}} = 0.30; \pi_{\text{tox}} = 0.40$;
- efficacy/no toxicity hypothesis: $\pi_{\text{eff}} = 0.50; \pi_{\text{tox}} = 0.30$;
- 4 and 5 doses explored;
- interim analysis at 35 patients per arm and final analysis at 70 patients per arm;
- maximum FWER = 10%.

Scenarios of simulation are displayed in figure S8.

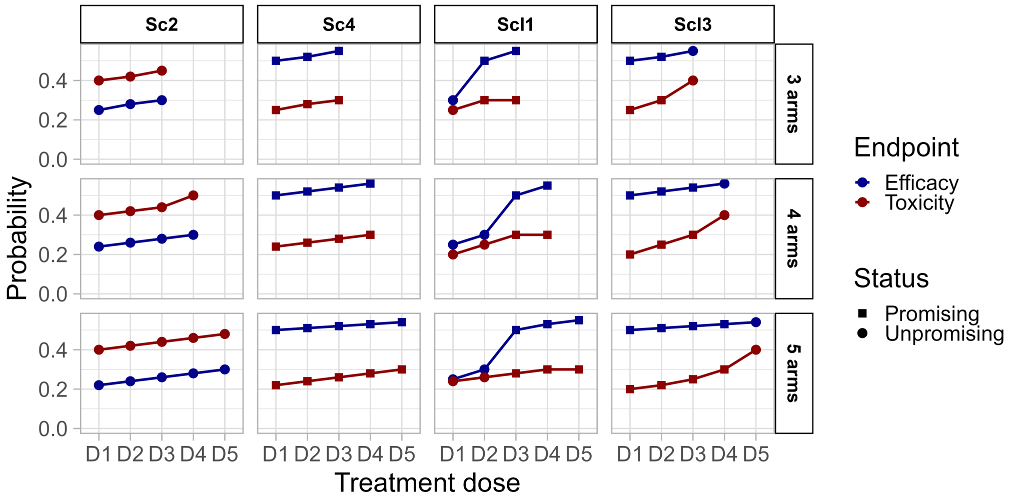

Figure S8: Sensitivity scenarios with 4 and 5 arms (3 arms is the main simulation in the main manuscript)

Overall, the results indicated that the risk of false positives in scenario 2 decreased as the number of studied arms increased, which is consistent with the fact that the FWER was not changed. Power in scenario 4 showed a similar pattern with increasing arms. In scenario I3, results were comparable across different numbers of arms, whereas in scenario I1, for logistic regression methods, the false positive rate for dose 2 with 4 and 5 arms was higher than for dose 1 with 3 arms, which were equivalent doses. Results are presented in figures S9 to S12.

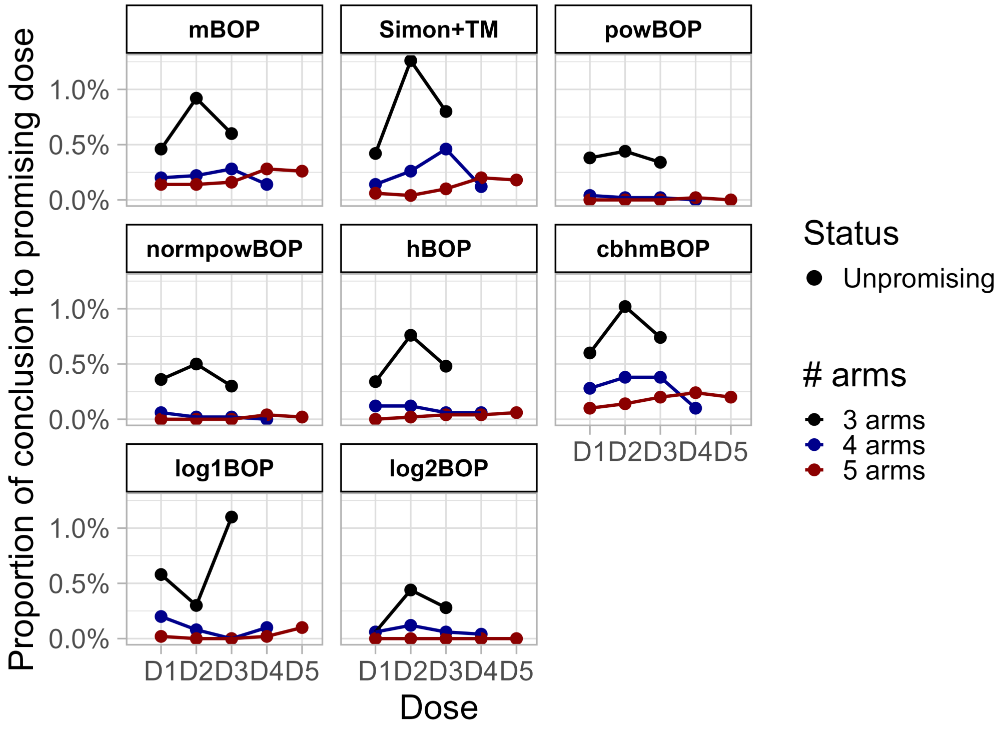

Figure S9: Proportion of conclusion in favor of a promising dose in each arms for each design in scenario 2 with 3, 4 and 5 doses explored

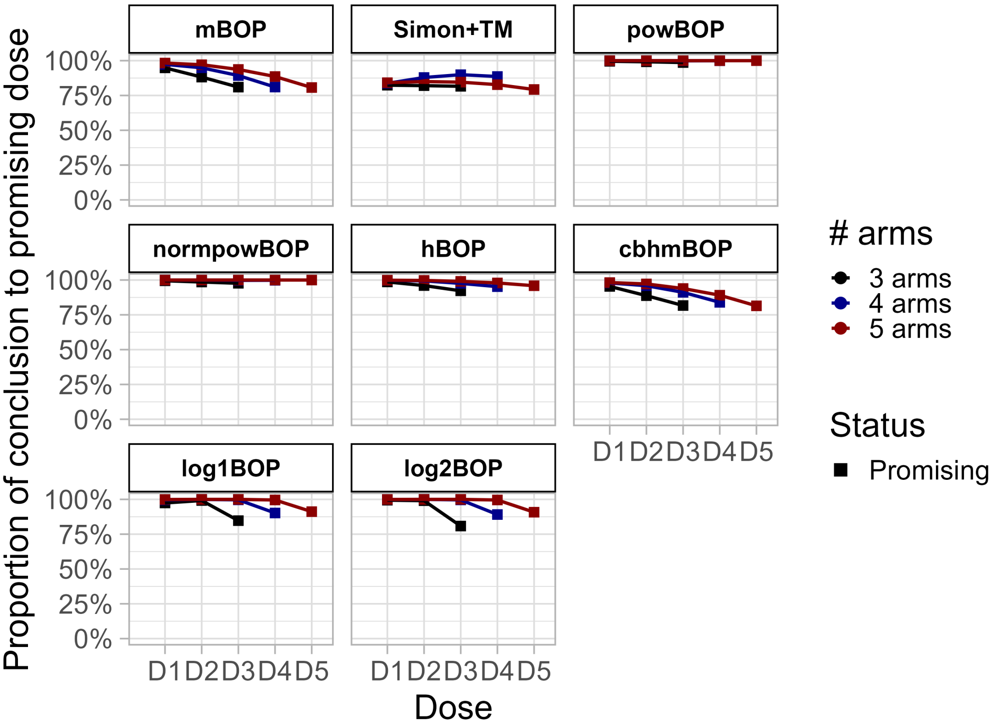

Figure S10: Proportion of conclusion in favor of a promising dose in each arms for each design in scenario 4 with 3, 4 and 5 doses explored

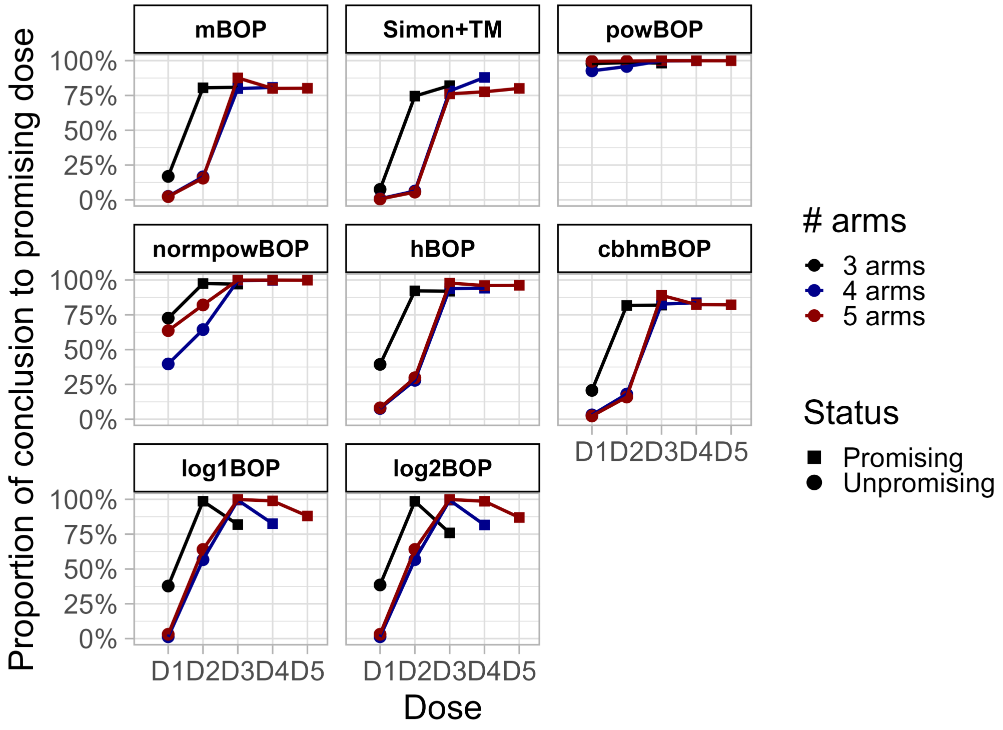

Figure S11: Proportion of conclusion in favor of a promising dose in each arms for each design in scenario I1 with 3, 4 and 5 doses explored

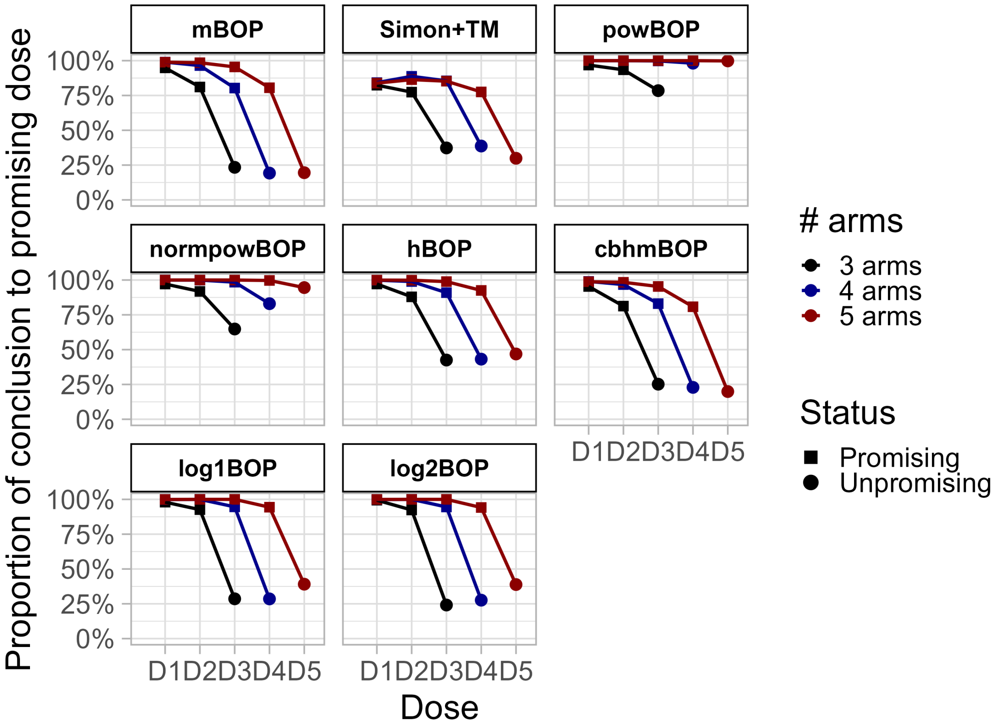

Figure S12: Proportion of conclusion in favor of a promising dose in each arms for each design in scenario I3 with 3, 4 and 5 doses explored

6 Sensitivity to priors

These sensitivity analyses were performed using the same settings as the main analysis.

6.1 hBOP

The general form of the models (efficacy and toxicity) is the following:

$$\begin{aligned}(x_1, \ldots, x_K) &\sim Binomial((n_1, \ldots, n_K), (p_1, \ldots, p_K)) \\ logit((p_1, \ldots, p_K)) &\sim \mathcal{N}(\mu, \sigma^2) \\ \mu &\sim \mathcal{N}(m_1, s_1^2) \\ \sigma &\sim \mathcal{HN}(0, s_2^2)\end{aligned}$$

Table S3: Sensitivity analysis parameters: priors for hBOP (in bold first line the change relative to the main analysis). When values are different from efficacy and toxicity, they are presented as efficacy/toxicity.

Model	m_1	s_1	s_2
hBOP	logit(inefficacy/toxicity hypothesis)	2.5	1
H1_1	**logit(efficacy/no toxicity hypothesis)**	2.5	1
H1_2	**logit(0.50)**	2.5	1
H2_1	logit(inefficacy/toxicity hypothesis)	**1.94/1.78**[*]	1
H2_2	logit(inefficacy/toxicity hypothesis)	**10**	1
H3_1	logit(inefficacy/toxicity hypothesis)	2.5	**5**
H3_2	logit(inefficacy/toxicity hypothesis)	2.5	**0.5**

[*] These correspond to weakly informative priors that corresponds to approximately one-observation ESS (Neuenschwander B, Wandel S, Roychoudhury S, Bailey S. Robust exchangeability designs for early phase clinical trials with multiple strata. Pharmaceutical statistics. 2016 Mar;15(2):123-34.).

Models H1_1, H1_2, H2_1 and H2_2 performed similarly to hBOP. H3_1 and H3_2 had different results: in scenarios I1 and I3, H3_1 was more conservative and H3_2 less conservative than mBOP; and in scenario 2, H3_1 was less conservative and H3_2 more conservative than mBOP. This could reflect the well-known caveat of hierarchical modeling: parameter σ is difficult to estimate, especially with low number of groups.

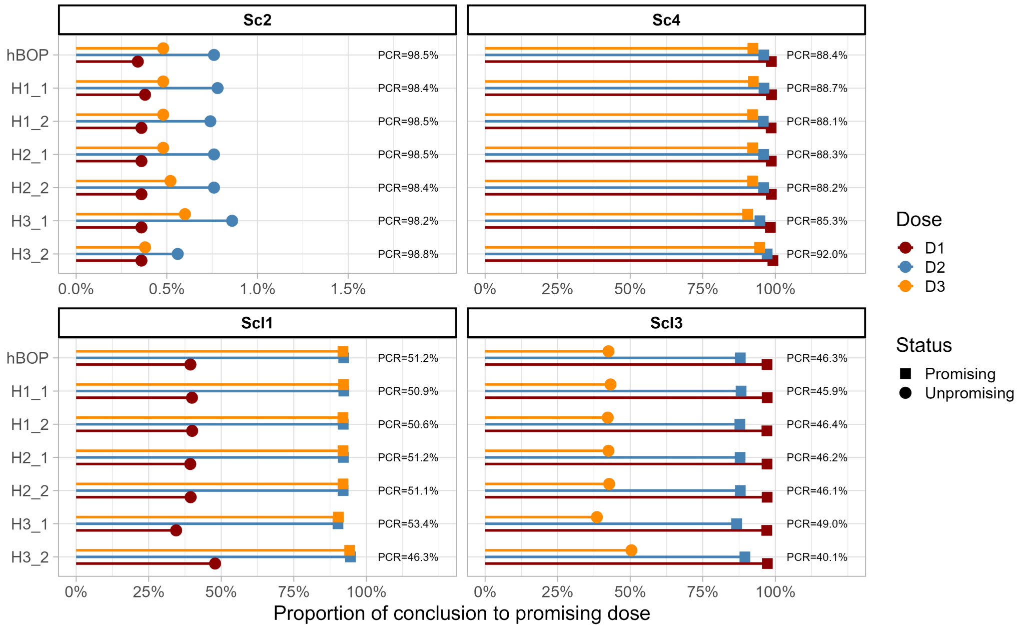

Figure S13: Proportion of conclusion to a promising dose in each arms for hBOP and its variations on the priors

6.2 cbhmBOP

The general form of the models (efficacy and toxicity) is the following:

$$
\begin{aligned}
(x_1, \ldots, x_K) &\sim Binomial((n_1, \ldots, n_K), (p_1, \ldots, p_K)) \\
logit((p_1, \ldots, p_K)) &\sim \mathcal{N}(\mu, \sigma^2) \\
\mu &\sim \mathcal{N}(m_1, s_1^2) \\
\sigma &= \sqrt{e^{a+b\times log(T)}}
\end{aligned}
$$

with T the χ^2 test statistic of homogeneity.

Table S4: Sensitivity analysis parameters: priors for cbhmBOP (in bold first line the change relative to the main analysis). When values are different from efficacy and toxicity, they are presented as efficacy/toxicity.

Model	m_1	s_1
cbhmBOP	logit(inefficacy/toxicity hypothesis)	2.5
C1_1	**logit(efficacy/no toxicity hypothesis)**	2.5
C1_2	**logit(0.50)**	2.5
C2_1	logit(inefficacy/toxicity hypothesis)	**1.94/1.78***
C2_2	logit(inefficacy/toxicity hypothesis)	**10**

* These correspond to weakly informative priors that corresponds to approximately one-observation ESS(Neuenschwander B, Wandel S, Roychoudhury S, Bailey S. Robust exchangeability designs for early phase clinical trials with multiple strata. Pharmaceutical statistics. 2016 Mar;15(2):123-34.).

Results are similar for all variations of the prior.

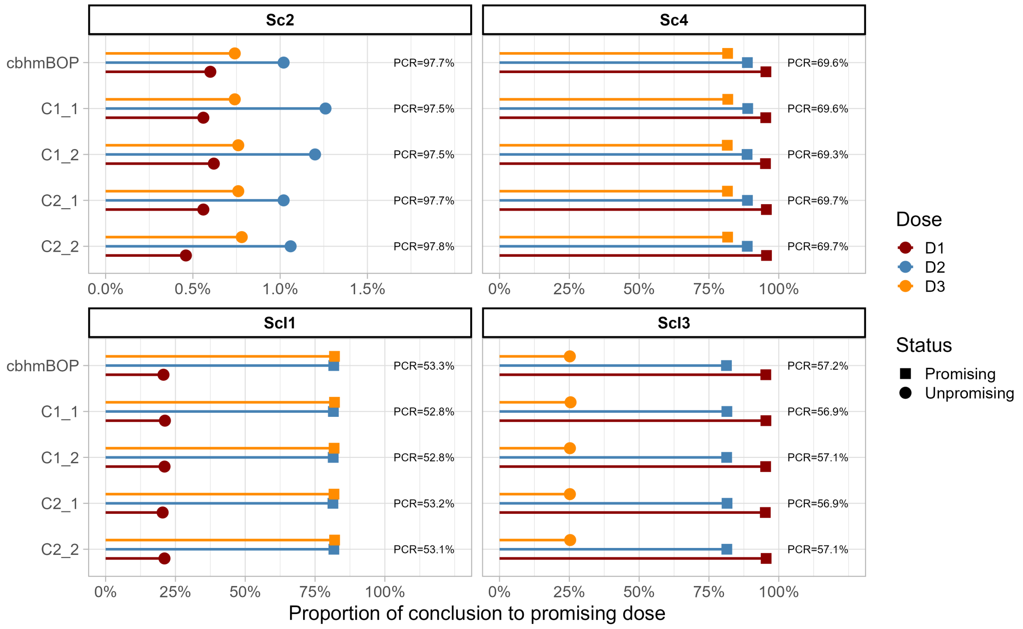

Figure S14: Proportion of conclusion in favor of a promising dose in each arms for cbhmBOP and its variations on the priors

6.3 log1BOP

The general form of the models (efficacy and toxicity) is the following:

$$logit(p_k) = \alpha + \beta \frac{d_k}{d*}$$
$$\alpha \sim \mathcal{N}(m_1, s_1^2)$$
$$\beta \sim \mathcal{N}(m_2, s_2^2)$$

Table S5: Variations of the priors studied for log1BOP (in bold first line the change relative to the main analysis). When values are different from efficacy and toxicity, they are presented as efficacy/toxicity.

Model	m_1	s_1	m_2	s_2
log1BOP	logit(inefficacy/toxicity hypothesis)	2.5	log(1.52)/log(1.25)	2.5
L1_1	**logit(efficacy/no toxicity hypothesis)**	2.5	log(1.52)/log(1.25)	2.5
L1_2	**logit(0.50)**	2.5	log(1.52)/log(1.25)	2.5
L2_1	logit(inefficacy/toxicity hypothesis)	**1**	log(1.52)/log(1.25)	2.5
L2_2	logit(inefficacy/toxicity hypothesis)	**10**	log(1.52)/log(1.25)	2.5
L3_1	logit(inefficacy/toxicity hypothesis)	2.5	log(1.52)/log(1.25)	**1**
L3_2	logit(inefficacy/toxicity hypothesis)	2.5	log(1.52)/log(1.25)	**10**
L4_1	logit(inefficacy/toxicity hypothesis)	2.5	**log(1)**	2.5
L4_2	logit(inefficacy/toxicity hypothesis)	2.5	**log(2.7)**	2.5
L5_1	logit(inefficacy/toxicity hypothesis)	2.5	**log(1)**	**1**
L5_2	logit(inefficacy/toxicity hypothesis)	2.5	**log(2.7)**	**1**

Results for log1BOP, L1_1, L1_2, L2_2, L3_1, L3_2, L4_1, L4_2 and L5_2 were similar. L5_1 had a slight increase in false positive rate in scenario I1, but L2_1 had an increase in false

positive rate in scenarios I1 and I3. This suggests that giving informative priors could lead to different results relative to how the prior is centered. Thus we advise to give weakly informative priors to the Bayesian logistic model.

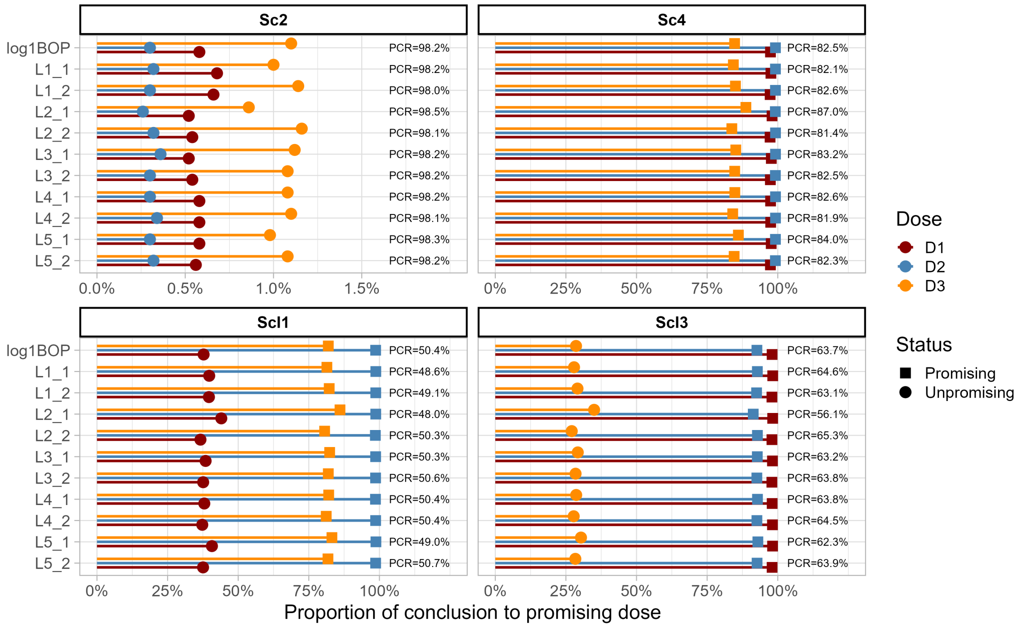

Figure S15: Proportion of conclusion in favor of a promising dose in each arms for log1BOP and its variations on the priors

7 Sensitivity analysis

Logistic model

Denote S_i the skeleton at dose i: $S_i = \frac{ln(\frac{\pi_i}{1-\pi_i})-\alpha}{\beta}$. For the computation of the skeleton, we used $\alpha = 3$ and $\beta = 1$. The model was the following (similar to log1BOP):

$$logit(p_k) = \alpha + \beta S_k$$
$$\alpha \sim \mathcal{N}(logit(H_0), 2.5^2)$$
$$\beta \sim \mathcal{N}(0.42/0.22, 2.5^2)$$

In the following, we further considered 2 models:

- crmunfixed, which is the 2-parameter logistic regression ;
- crmfixeda3, which is the same model but with α fixed at 3 resulting in a one-parameter model.

Power model

The skeleton at dose i is $S_i = \pi_i^{e^{-\beta}}$. For computations, we used β of 0 and 1. The model is the following:

$$y_k \sim \text{Bernoulli}(S_i^{e^{\beta}})$$
$$\beta \sim \mathcal{N}(0, 5^2)$$

We examined 2 models:

- crmpowbop with $\beta = 0$ to compute the skeleton;
- crmpowbop2 with $\beta = 1$ to compute the skeleton.

Optimism of the skeleton

We tested:

- an optimistic skeleton (denoted "opti" in figure S16: efficacy of (0.50, 0.55, 0.60) for the 3 doses and toxicity of (0.20, 0.25, 0.30) for the 3 doses;
- a skeptical skeleton (denoted "pess" in figure S16: efficacy of (0.25, 0.27, 0.30) for the 3 doses and toxicity of (0.40, 0.45, 0.50) for the 3 doses;
- a skeleton going from H_0 to H_1 (denoted "main" in figure S16: efficacy of (0.30, 0.40, 0.50) for the 3 doses and toxicity of (0.30, 0.35, 0.40) for the 3 doses.

Results

In scenario 2, all CRM-type models were more conservative than mBOP and, in scenario 4, power was improved, particularly for one-parameter logistic model (crmunfixed) whichever the skeleton. For scenarios I1 and I3, the models lead to an inflated false positive rate in dose 1 of scenario I1 and dose 3 of scenario I3.

Results mostly depended on the dose skeleton, corresponding to prior guesses on the efficacy and toxicity rates at each of the 3 candidate dose levels, emphasizing the importance of the skeleton in this phase II randomized setting, including decision rules on the estimated outcomes rates.

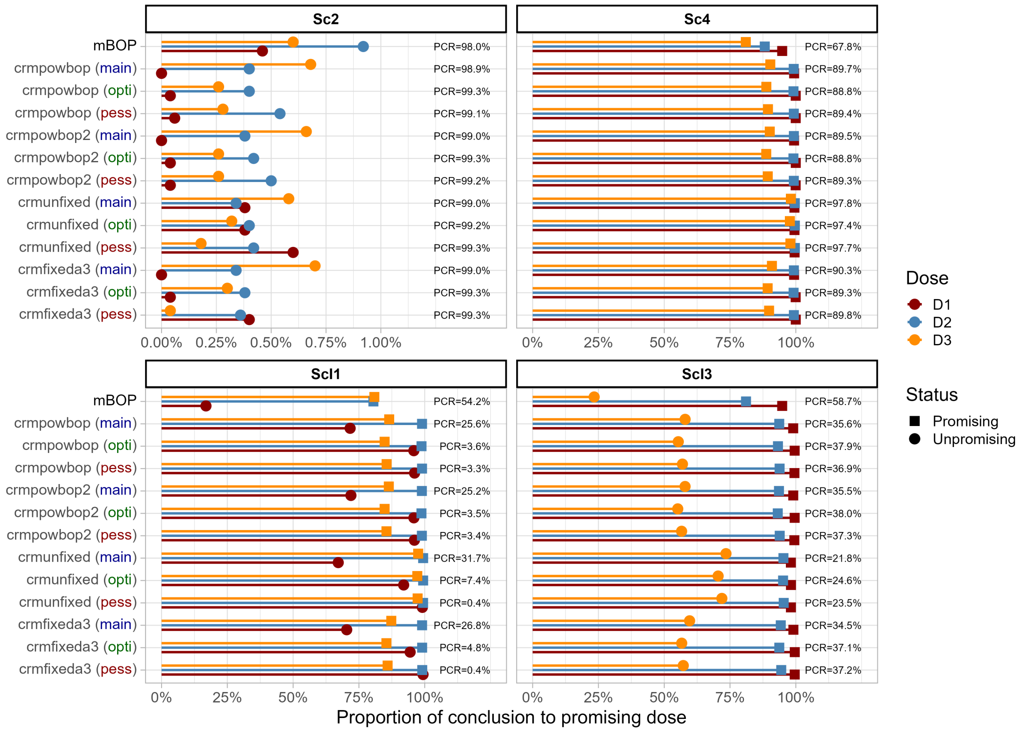

Figure S16: Proportion of conclusion in favor of a promising dose in each arms for the crm-type models in scenarios 2, 4, I1 and I3. (mBOP has been added as a comparator)
